# Handling the heat: ocean acidification mitigates the effects of marine heatwaves on *Posidonia oceanica* seedlings

**DOI:** 10.1093/jxb/eraf276

**Published:** 2025-06-24

**Authors:** Jessica Pazzaglia, Lazaro Marín-Guirao, Luca Ambrosino, Katia Pes, Monya Costa, Isabel Barrote, João Silva, Gabriele Procaccini

**Affiliations:** Department of Integrative Marine Ecology, Stazione Zoologica Anton Dohrn, Villa Comunale, Naples 80121, Italy; National Biodiversity Future Center, Palermo 90133, Italy; Centro Oceanográfico de Murcia, Instituto Español de Oceanografía (IEO, CSIC), San Pedro del Pinatar, Murcia 30740, Spain; Department of Research Infrastructure for Marine Biological Resources, Stazione Zoologica Anton Dohrn, Villa Comunale, Napoli 80121, Italy; Excellence Cluster Cardio-Pulmonary Institute (CPI), Justus-Liebig-University, Giessen 35392, Germany; Greencolab - Associação Oceano Verde, Universidade do Algarve, Faro 8005-139, Portugal; Faculdade de Ciências e Tecnologia, Campus de Gambelas, Universidade do Algarve, Faro 8005-139, Portugal; Centre of Marine Sciences (CCMAR/CIMAR LA), Campus de Gambelas, Universidade do Algarve, Faro 8005-139, Portugal; Centre of Marine Sciences (CCMAR/CIMAR LA), Campus de Gambelas, Universidade do Algarve, Faro 8005-139, Portugal; Department of Integrative Marine Ecology, Stazione Zoologica Anton Dohrn, Villa Comunale, Naples 80121, Italy; National Biodiversity Future Center, Palermo 90133, Italy; Lawrence Berkeley National Laboratory, USA

**Keywords:** Acclimation, acidification, antioxidant response, energetic metabolism, heatwave, multiple stressors, physiology, seagrasses, transcriptomics

## Abstract

Ocean acidification and marine heatwaves are key drivers of marine ecosystem changes that can interact with one another and influence marine organisms. Seagrasses, including the long-lived *Posidonia oceanica* that is endemic to the Mediterranean Sea, are widely distributed along coastal habitats, forming highly valuable underwater meadows. The germination and survival of the early life stages of *P. oceanica* are strongly affected by environmental changes. To assess the impact of warming and acidification on its future, we conducted a multifactorial experiment in which *P. oceanica* seedlings were grown under ocean acidification conditions for 6 months and then exposed to a seawater warming event. Seedling performance was investigated by analysing photo-physiology, antioxidant capacity, energetic metabolism, and transcriptomic profiles. A weighted gene correlation network analysis was used to integrate phenotypic plant traits with transcriptomic results to identify central genes involved in plant responses to ocean acidification and temperature exposure. Results demonstrated that prolonged ocean acidification exposure enhances *P. oceanica* seedling resilience to marine heatwaves. Specifically, seedlings regulated their antioxidant systems and transcriptomic machinery to better cope with thermal stress. Under current CO_2_ concentrations, elevated temperatures induced stress in *P. oceanica* seedlings, impacting photosynthesis and respiration. However, ocean acidification could mitigate the impact of warming in the future, enhancing the resilience to global stressors of *P. oceanica*.

## Teaser

Ocean acidification enhances seagrass (*Posidonia oceanica*) seedling resilience to marine heatwaves by modulating antioxidant defenses, metabolism, and gene expression, potentially mitigating climate change impacts on seagrass ecosystems.

## Introduction

Carbon emissions from human activities are threatening coastal marine ecosystems worldwide. Over the last 250 years, oceans have become a carbon dioxide (CO_2_) sink, taking up between 20% and 30% of total emissions of atmospheric CO_2_ (Bindoff *et al*., 2019). The projected time of emergence, which refers to when a climate change signal becomes distinguishable from natural variability, placed ocean acidification (OA) as the primary driver of marine ecosystem changes, followed by ocean warming, which is also correlated with the increase of CO_2_ in the atmosphere (IPCC, 2013). Longer and more intense anomalous events of seawater warming (i.e. marine heat waves, MHWs) are more frequent, and their duration (annual MHW days) globally increased by 54% during the last decades (Oliver *et al.*, 2018). Considering also the emerging local stressors in coastal habitats (e.g. eutrophication), 97.7% of the oceans are globally affected by more than one stressor that potentially interacts with others, exacerbating the overall impact on individuals, populations, and ecosystems (Halpern *et al*., 2015). The seagrass species *Posidonia oceanica* is endemic to the Mediterranean Sea, where it grows along the coasts of the whole basin, except for the southeastern region, showing a clear genetic structure among populations distributed along bathymetrical and latitudinal gradients (Jahnke *et al*., 2019; Procaccini *et al*., 2023). It is a key foundation species hosting 25% of the total biodiversity of the Mediterranean basin (Relini, 2008), storing 14.6–36.9 million t C_org_ in the matte system (Monnier *et al*., 2022), regulating biogeochemical cycles, and protecting coasts from erosion (Ondiviela *et al*., 2014). Distinct populations also show differential transcriptomic responses when exposed to simulated sea warming experiments (Marín-Guirao *et al*., 2017, 2019; Pazzaglia *et al*., 2022b). Extensive *P. oceanica* meadows can counteract possible adverse effects of acidification by modifying the pH in the water column (Hendriks *et al*., 2014) while sequestering and immobilizing carbon in their rhizomes.

Seagrasses are expected to benefit from high levels of CO_2_ in seawater, increasing carbon fixation and thus photosynthesis (Koch *et al*., 2013) and simultaneously reducing photorespiration. In the context of climate change, rising temperature alters the carbon balance in the plant (Nguyen *et al*., 2021) and promotes the accumulation of reactive oxygen species (ROS) (Tutar *et al*., 2017). Although the isolated effects of OA and temperature on seagrass performance have been explored considering photo-physiological responses (Apostolaki *et al*., 2014; Pedersen *et al*., 2016; Piro *et al*., 2020; Rubio *et al*., 2020; Deguette *et al*., 2022), energetic metabolism (Beca-Carretero *et al*., 2018; Pacella *et al*., 2018), epiphyte communities (Cox *et al*., 2015; Ramajo *et al*., 2019; Lee *et al*., 2022), and gene expression (Olivé *et al*., 2017; Ruocco *et al*., 2017), there are only a few studies reporting their combined effects (*Zostera marina*, Repolho *et al.*, 2017; Zayas-Santiago *et al*., 2020; *Thalassia hemprichii*, Listiawati and Kurihara, 2021; *Cymodocea serrulata*, Viana *et al.*, 2023). Several studies have revealed inter- and intraspecific responses of adult seagrass plants to various stressors (York *et al*., 2013; Dattolo *et al*., 2014; Ontoria *et al*., 2019; Pazzaglia *et al*., 2020, 2022b; Ruocco *et al*., 2020; Guerrero-Meseguer *et al*., 2020a; Kaldy *et al*., 2022), while little is known about the effect of multiple stressors on the early life stages (*P. oceanica*, Guerrero-Meseguer *et al*., 2017, 2020b; Hernán *et al*., 2017; Pereda-Briones *et al*., 2019; Rinaldi *et al*., 2023). For instance, only Lowell *et al.* (2021) and Hernán *et al.* (2016) have investigated the OA effect on *Z. marina* seedlings, and no one has studied the impact of both long-term OA and marine heat wave (MHW) exposure.

Seagrass genotypes have large phenotypic plastic properties that have favored species diversification and the colonization of heterogeneous environments (Pazzaglia *et al*., 2021; Sandoval-Gil *et al*., 2023). This is the case of *P. oceanica*, which occurs from 1 m to 40 m in depth and colonizes heterogeneous environments where environmental features (i.e. salinity, turbidity, temperature, and light conditions) show clear gradients (Procaccini *et al*., 2017). Seagrasses also have different colonization strategies, ranging from short-lived plants with small seeds that germinate close to the parent meadows (*Halophyla* sp., Gu *et al.*, 2022), to long-living ones that form buoyant fruits with large dispersal capacity (*Posidonia* sp., Guerrero-Meseguer *et al.*, 2018). In the context of climate change, seagrasses can respond in different ways to unfavorable conditions (Nguyen *et al*., 2021). For instance, flowering events observed in *P. oceanica* meadows were positively correlated with mean summer sea surface temperature (SST) (Stipcich *et al*., 2024), and were identified as a stress response with potential adaptive consequences for the species (Ruiz *et al*., 2018; Marín-Guirao *et al*., 2019). Nevertheless, little is known about the capability of seedlings to establish and grow in a future environmental scenario, under increased OA and MHW frequency.

Thus, exploring mechanisms that favor seagrass adjustments to stressful conditions (i.e. acclimation) is important for understanding their biology and predicting future responses to environmental changes. Most studies performed on *P. oceanica* seedlings have analysed stress responses at morphological, physiological, and biochemical levels (Hernán *et al*., 2016; Guerrero-Meseguer *et al*., 2017; Pazzaglia *et al*., 2022a; Stipcich *et al*., 2022), while whole-genome transcription analysis is completely unexplored (but see Pazzaglia, *et al*., 2022b). While photo-physiological and metabolic analyses are valuable tools for assessing plant performance in response to stress exposure, molecular approaches, such as RNA sequencing and DNA methylation, can quantitatively analyse changes in gene expression and methylation at a specific time point and state, revealing complex regulatory networks (Anderson and Mitchell-Olds, 2011). It is widely known that multilevel crosstalk mechanisms based on gene transcripts and epigenetics regulate phenotypic changes in the presence of environmental shifts, forming a complex machine that promotes fast reactions and adjustments to newly imposed conditions (Xi *et al.*, 2020; Pazzaglia *et al.*, 2021; Hansen *et al.*, 2022). Under stressful conditions, genes cooperate and are modulated in clusters (e.g. co-expression) to activate regulatory strategies (Williams and Bowles, 2004). Co-expressed gene clusters can be functionally similar or have common biological regulatory roles. Novel bioinformatics applications, like the weighted gene co-expression network analysis (WGCNA), build correlation networks of clustered genes (i.e. modules) that are represented by module eigengene values and correlate modules to phenotypic traits (Langfelder and Horvath, 2008). The power of this analysis is to identify a cluster of genes differentially expressed and significantly involved in regulating a phenotypic trait under stress exposure (Yu *et al*., 2020). In plants, the integration of phenotypic responses with transcriptomic has been a useful approach for identifying pools of genes directly involved in the regulation of the stress response (Amrine *et al*., 2015; Reimer *et al*., 2021; Riccio-Rengifo *et al*., 2021; Ju *et al*., 2023). A similar analysis was recently applied also in *P. oceanica* adult plants to search for early warning indicators of stress signals derived from temperature and nutrient stressors (Santillán-Sarmiento *et al*., 2023). In that case, shoot survival was integrated with transcriptomic profiles through the WGCNA allowing the identification of several transcripts involved in stress-related biological processes.

In the present study, we aimed to explore how prolonged exposure to OA affects the ability of *P. oceanica* seedlings to cope with MHW, simulating a predicted future scenario of OA and MHW co-occurrence. For this purpose, we performed a multifactorial experiment in which *P. oceanica* seedlings were grown under OA conditions for 6 months and then exposed to an anomalous seawater warming event. Phenotypic traits such as photo-physiology, antioxidant capacity, and energetic metabolism were analysed and integrated with transcriptomic data by applying the WGCNA to explore potential interactive effects between OA conditions and temperature increase.

## Materials and methods

### Experimental design and seedlings collection

**Fig. 1.**
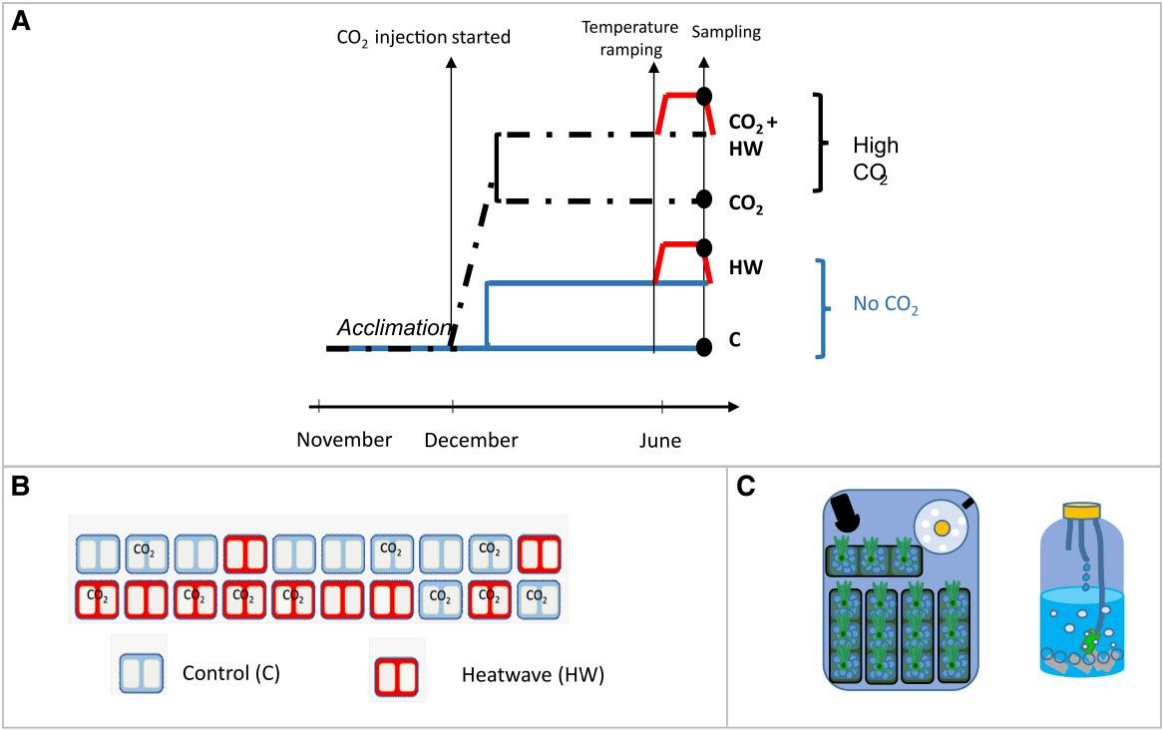
Experimental design and treatment distribution. (A) Layout of the experimental setup. (B) Distribution of independent aquaria with randomly assigned CO₂ and temperature treatments. Quadrats represent tanks under control temperature conditions, and tanks subjected to heat wave stress. (C) Close-up of a single 20 liter aquarium showing marble-filled seed pots collected during the experiment, a water circulation pump, and the gas bubbling system. Detail of the gas bubbling apparatus is also shown, through which CO₂-enriched air was introduced into the aquaria in the CO_2_ enriched treatments.

*Posidonia oceanica* seeds collected in June 2019 along the coasts of Marsala (West Sicily) were left to germinate and grow as described in Pazzaglia *et al*. (2022a). By the end of October, seedlings were shipped by plane in thermic flasks to the Centre of Marine Sciences (CCMAR) in Faro, Portugal, where they were immediately transplanted in individual small seed pots (5×5×6 cm) filled with marbles and allocated randomly in 20 fully independent 20 liter experimental aquaria (15 seedlings per aquarium) (Fig. 1B), installed in an indoor mesocosm facility. The system was an open-circuit type, with each aquarium fitted with an aeration unit and a small water circulation pump (Fig. 1C). Seawater was pumped from a coastal lagoon (Rio Formosa) adjacent to CCMAR marine station and pre-filtered (sand filters, in-line cartridge filters and a 16 W UV filter), before entering the mesocosm facility. Long-term monitoring data from the coastal lagoon indicate relatively stable conditions in term of salinity and alkalinity that consist of 34–37 parts per thousand and 2450–2600 μmol kg⁻¹, respectively. At the beginning of the acclimation period, the photoperiod was set to 10 h:14 h (light: dark). The photoperiod was changed periodically during the experiment following the natural number of light: dark hours.

After 6 weeks of acclimation to mesocosm conditions under normal pCO_2_, ambient water temperature, and 55.6±3.3 μmol photons m^−2^ s^−1^ of light intensity, pCO_2_ was gradually increased in half of the tanks until a maximum of 750–900 ppm (water pH 7.790±0.062) (Fig. 1A). CO_2_-enriched air was continuously prepared in a large-volume premix tank (4000 liters) where industrial grade CO_2_ was blended with air to obtain the target pCO_2_. The premix tank was controlled via direct analysis of CO_2_ using an infrared gas analyser (WMA-4, PPSystems) coupled to a PID controller (PID330; Tempatron) that operated a solenoid valve to regulate the CO_2_ flush into the premix tank. The CO_2_-enriched air was pressure-pumped into a distribution pipe and readily available on tap in the mesocosm facility. In the tanks assigned to the high-CO_2_ treatment, this enriched blend was bubbled into the individual aeration units, from where CO_2_-enriched water circulated through the aquarium. The effectiveness of the system in attaining the desired CO_2_ levels and a proper homogenization of the conditions throughout the aquarium was thoroughly tested beforehand. The remaining tanks were kept under ambient temperature and pCO_2_ (water pH 8.186±0.050). Seedlings were grown under high CO_2_ and control conditions for 6 months (until mid-June).

In June, the water temperature in the aquaria was set and stabilized at 24 °C, reflecting the average temperature of the water entering the system during the preceding month. After 1 week at 24 °C, a heatwave was simulated in half of the high CO_2_ aquaria (five aquaria) and half of the control CO_2_ aquaria (five aquaria) by gradually increasing temperature along a heating ramp (1 °C d^−1^) up to 32 °C. Throughout the entire acclimation and experimental period, the aquaria water temperature and pH were monitored daily using HOBO Pendant data loggers (Onset Corp.) and a Thermo Scientific Orion 8103SC pH meter, respectively. Water temperature was additionally monitored daily (Roth digital thermometer, Hanna) in each aquarium at the warmest time of the day (between 14.00 and 16.00 h). Irradiance was measured periodically in every tank (LI-192+LI-250, Li-Cor). pCO2 was monitored continuously with a non-dispersive infrared gas analyser (WMA-4, PP Systems, UK) coupled to a gas exchange column (Mini-Module membrane contactor, Celgard, USA). Salinity (CO310 conductivity meter, VWR) was also measured daily, between 11.00 and 13.00 h, in all tanks.

After 5 d at 32 °C, leaf samples were collected from the seedlings under the different experimental conditions (*n*=5): control (C, *T*=24 °C, current CO_2_); high CO_2_ (CO_2_, *T*=24 °C, 700 ppm CO_2_); heatwave (HW, *T*=32 °C, ambient CO_2_); heatwave under high CO_2_ (CO_2_+HW, *T*=32 °C, 700 ppm CO_2_). Leaves were cleaned of epiphytes, rinsed with distilled water, blotted dry, and stored at −80 °C until analysis for photosynthetic pigments, antioxidant capacity, adenylate charge, soluble sugars, and starch content. The youngest leaves (two- and three-ranked leaves) were collected per each treatment for transcriptomic analysis, gently cleaned of epiphytes, and entirely submerged in RNAlater© (Thermo Fisher Scientific).

### Photo-physiological assessment

The potential quantum yield of photosystem II (*F*_v_/*F*_m_) was measured every 2 weeks during the experimental periods (Supplementary Fig. S1) in one plant per tank at the middle section of the youngest or second-youngest leaf using a pulse amplitude-modulated fluorometer (Diving-PAM, Heinz Walz, Effeltrich, Germany). *F*_v_/*F*_m_ was measured after the dark period and before switching on the lights. Photosynthesis–irradiance curves (*P–I* curves) were measured in *P. oceanica* leaves of all the aquaria at the end of the heatwave (*n*=5). The setup comprised five independent chambers filled with water from the aquaria and sealed with a Petri dish containing an optical O_2_ sensor (Presens Spot PS). The water inside each incubation chamber was maintained at the same temperature and pH as in the aquaria by a closed-circuit thermostatic water-bath temperature controller (Julabo HC, Julabo Labortechnik, Seelbach, Germany); magnetic stirrers ensured water homogenization inside the incubation chambers. Five LED lamps provided the light, and the nine different light intensities plus dark were achieved by using various combinations of neutral-density filters. Leaf samples (*n*=5) were collected, cleaned of epiphytes, and immediately placed inside the incubation chambers in the dark. After a 20 min dark acclimation period, O_2_ concentration (μmol l^−1^) was measured in each chamber with a Microx 4 PreSens Optode (Regensburg, Germany). Then, a further 15 min dark incubation was carried out to assess dark respiration. The light was then switched on, and after a 15 min incubation under the lower light intensity, O_2_ concentration (μmol l^−1^) was measured in each chamber. The same procedure was followed for increasing light intensity of photosynthetically active radiation (PAR) within the range 0–1252 μmol l^−1^ photons m^−2^ s^−1^. Net photosynthetic (NP) and dark respiration rates were calculated as follows:


$$(({\left[O_{2}\right]}_{f}\text{–}{\left[O_{2}\right]}_{i})\times V)/T/A$$

where [O_2_]_f_ is the final O_2_ concentration (μmol l^−1^), [O_2_]_i_ is the initial O_2_ concentration (μmol l^−1^), *V* is the volume of water in each chamber (l), *T* is the incubation time (h), and *A* is the leaf area (m^2^). During the measurements, O_2_ saturation levels were periodically checked, and incubation time was adjusted to avoid O_2_ supersaturation in the chambers. *P–I* curves were fitted with the equation model of Smith (1936) and Talling (1957) using SigmaPlot (version 14.0, 2017, Systat Software Inc.), and the maximum photosynthetic rate (*P*_m_, μmol O_2_ m^−2^ h^−1^) was calculated.

The photosynthetic pigments, chlorophyll *a*, chlorophyll *b*, and total carotenoids (Car), were extracted from 200 mg of frozen leaf tissue, which was powdered in liquid nitrogen with sodium ascorbate under dim light. Extraction was performed using 5 mL of pure acetone neutralized with calcium carbonate (Costa *et al*., 2021). The extracts were sequentially filtered with a 5.0 μm LS membrane and 0.2 μm polytetrafluoroethylene (PTFE) hydrophobic filter. The photosynthetic pigments were quantified spectrophotometrically (Beckman Coulter DU-650 spectrophotometer) by reading the extract absorbances at 470, 644.8, and 661.6 nm. The concentration of photosynthetic pigments was calculated according to Lichtenthaler and Buschmann (2001).

### Antioxidant capacity, oxidative stress, and energy metabolism

The leaf antioxidant capacity was assessed by quantifying oxygen radical absorbance capacity (ORAC) as in Costa *et al*. (2021) after Re *et al*. (1999) and in Gillespie *et al*. (2007). Briefly, 100 mg of frozen leaf tissue was powdered in liquid nitrogen, suspended in 3 ml of hydrochloric acid (HCl) 0.1 M, kept overnight under constant shaking at 4 °C, and centrifuged (4700*×g*, 4 °C, 30 min). The supernatant was used for ORAC assays.

For the ORAC assay, 150 μl of 8.2×10^−5^ mM fluorescein in 75 mM potassium phosphate buffer, pH 7.4, was added to 25 μl of extract, heated to 37 °C, and read in a Synergy TM4 multi-detection microplate reader (485 nm excitation filter, 20 nm bandpass, and 528 nm excitation filter, 20 nm bandpass). The reaction was initiated by adding 25 μl of freshly prepared 153 mM 2,2′-azobis (2-amidinopropane) (a lipophilic peroxyl radical generator). Results were expressed as Trolox® equivalents.

Energy metabolism was assessed by the quantification of the adenylate compounds ATP, ADP, and AMP, soluble sugars, and starch. ATP, ADP, and AMP were extracted from frozen leaves and roots according to Liu *et al.* (2006) and quantified by isocratic HPLC analysis as in Coolen *et al.* (2008) in an Alliance Waters 2695 separation module (Milford, MA, USA) with a Waters 2996 photodiode array detector and 150×4.6 mm, Kinetex 5μ C18 100 Å column (Phenomenex ®). The adenylate energy charge (AEC) was calculated as in Padinha *et al*. (2000):


AEC=(ATP+0.5×ADP)/(ATP+ADP+AMP)

Soluble sugars and starch were extracted from *P. oceanica* leaves and roots. Ten milligrams of freeze-dried leaf tissue was powdered in a ball mill (Retsch, MM300), extracted in ethanol 80% (10 min, 80 °C), and centrifuged (2000*×g*, 4 °C, 5 min). The supernatant was collected for soluble sugars and the pellet for starch quantification. Before starch quantification, the pellet was washed three times with distilled water (1 ml) and incubated at 100 °C for 10 min. Then, starch was hydrolysed by a 24 h incubation at 37 °C in an enzyme mix (4 U ml^−1^ α-amylase and 2.8 U ml^−1^ amyloglucosidase in sodium acetate 0.2 M, pH 4). After hydrolysis, the mixture was centrifuged (750*×g*, 4 °C, 5 min) and used for starch quantification. Soluble sugars and starch were quantified by mixing 1 ml of extract with 1 ml of 5% phenol and 5 ml of 95% sulfuric acid and reading absorbances at 450 nm and 750 nm (Beckman Coulter DU-650 spectrophotometer) and reported as total non-structural carbohydrates (TNC) in the leaf and root. Calibration was performed using several dilutions of glucose.

### RNA extraction and sequencing

Samples were stored overnight at 4 °C to let the RNAlater solution penetrate the tissue and then stored at −20 °C until RNA extraction. Total RNA was extracted from three replicates using the Aurum™ Total RNA Mini Kit (Bio-Rad). RNA purity and concentration were checked using a NanoDrop® ND-1000 spectrophotometer (Thermo Fisher Scientific) and 1% agarose gel electrophoresis, while RNA integrity was assessed by means of 2100 BioAnalyser (Agilent Technologies). Next-generation sequencing experiments were performed by Genomix4life S.R.L. (Baronissi, Salerno, Italy). Indexed libraries were prepared from 800 ng each purified RNA with Illumina Stranded mRNA Prep according to the manufacturer’s instructions. Libraries were quantified using the TapeStation 4200 (Agilent Technologies) and Qubit fluorometer (Thermo Fisher Scientific), then pooled such that each index-tagged sample was present in equimolar amounts, with a final concentration of the pooled samples of 5 nM. The pooled samples were subject to cluster generation and sequencing using an Illumina Novaseq 6000 System in a 2×151 paired-end format. The raw sequence files generated (.fastq files) underwent quality control analysis using FastQC (http://www.bioinformatics.babraham.ac.uk/projects/fastqc).

### Differentially expressed genes and functional annotation analysis

A cleaning step of the raw reads was performed using Trimmomatic (Bolger *et al*., 2014). Low-quality reads were discarded during the cleaning step, setting the minimum quality per read at a 20 phread score. All cleaned reads were then mapped on the reference genome of *P. oceanica* (Ma *et al*., 2024) using STAR aligner v.2.6.0c (default settings; Dobin *et al*., 2013). Reads count per gene for each replicate were performed using featureCounts software (Liao *et al*., 2014). A differentially expressed gene (DEG) analysis was carried out using DESeq2 (Love *et al*., 2014) and edgeR (Robinson *et al*., 2010), two R packages implementing two different statistical approaches. For each gene, the mean of the log2 fold change (log2FC) values obtained with DESeq2 and edgeR was retrieved, setting as parameters an false discovery rate ≤0.05, an adjusted *P*-value ≤0.05, and |log2FC|≥1.5. Differential gene expression profiles were calculated for the comparisons between all treatments (heatwave, CO_2_, and heatwave+CO_2_) and the control condition. Functional annotation of the reference transcriptome was carried out by scanning the Swiss-Prot database (Bateman, 2019) using the BLASTx software (Camacho *et al*., 2009), setting an E-value threshold of 1e^−3^ and getting only the best hit detected. For each best hit, the SwissProt function and the related Gene Ontologies (GO) were retrieved. A GO enrichment analysis for all the identified DEG datasets was carried out by Ontologizer software (Bauer *et al*., 2008), setting a *P*-value to ≤0.05 to identify significantly enriched functional terms.

### Weighted gene co-expression network analysis

Reads counts were filtered to remove genes with less than 10 counts in all the replicates, to remove low abundancies or noise from the analysis. Filtered counts for all conditions were used to generate co-expression networks using the weighted gene co-expression network analysis (WGCNA) package in R (Langfelder and Horvath, 2008). The adjacency matrix was constructed using a soft threshold power of 14 (Supplementary Fig. S2). To generate networks of co-expressed genes, the blockwiseModules function (Zhang and Horvath, 2005) was used to transform the adjacency values into topological overlap measure (TOM). The different modules were identified using the dynamic tree cut algorithm (Langfelder *et al*., 2008) setting a minimum cluster size (minModuleSize parameter) of 30 and a merging threshold function (mergeCutHeight parameter) of 0.25. To relate different studied traits with the gene networks, the MRs (the first principal component of each module) were correlated to 18 physiological variables. The corPvalueStudent function was selected to use a Student asymptotic *P*-value for correlation. Hub genes were identified within each module based on their module membership (MM) value, also known as eigengene-based connectivity (kME, intramodular connectivity; Fuller *et al*., 2007). This value represents the correlation between the expression level of a particular gene and the module eigengene (ME; Horvath and Dong, 2008). Genes with higher MM values are highly connected within a particular module (Fuller *et al*., 2011). A GO enrichment analysis for all the detected modules was carried out with Ontologizer software (Bauer *et al*., 2008), setting a *P*-value to ≤0.05 to identify significantly enriched co-expressed functional terms. The reads per kilobase of transcript per million mapped reads (RPKM) was considered for discussing the abundance level of key genes observed in the significant modules.

### Statistics

Plant trait responses [*F*_v_/*F*_m_, net photosynthesis, dark respiration, total photosynthetic pigments, gross photosynthesis, gross photosynthesis: dark respiration ratio (P:R ratio), ORAC, AEC, leaf TNC, root TNC] were analysed with two-way ANOVA to detect significant differences between treatments and control. The model included CO_2_ (with two levels: control and high) and temperature (HW, with two levels: control and high) as fixed factors. Since AEC and TNC were measured in leaves and roots, three-way ANOVA was applied to investigate significant differences between treatments and tissues, including CO_2_ (with two levels: control and high), temperature (HW, with two levels: control and high), and tissues (with two levels: leaf and roots) as fixed factors. Normality and homoscedasticity were checked using the Shapiro–Wilk test and Levene's test, and data were subsequently transformed where necessary. Tukey's HSD (honestly significant difference) test post-hoc comparison was used whenever significant differences (*P*<0.05) among treatments were detected. Statistical analyses were performed in R version 4.2.0 (R Core Team, 2021).

## Results

### Photo-physiological traits, respiration and pigment content

**Fig. 2.**
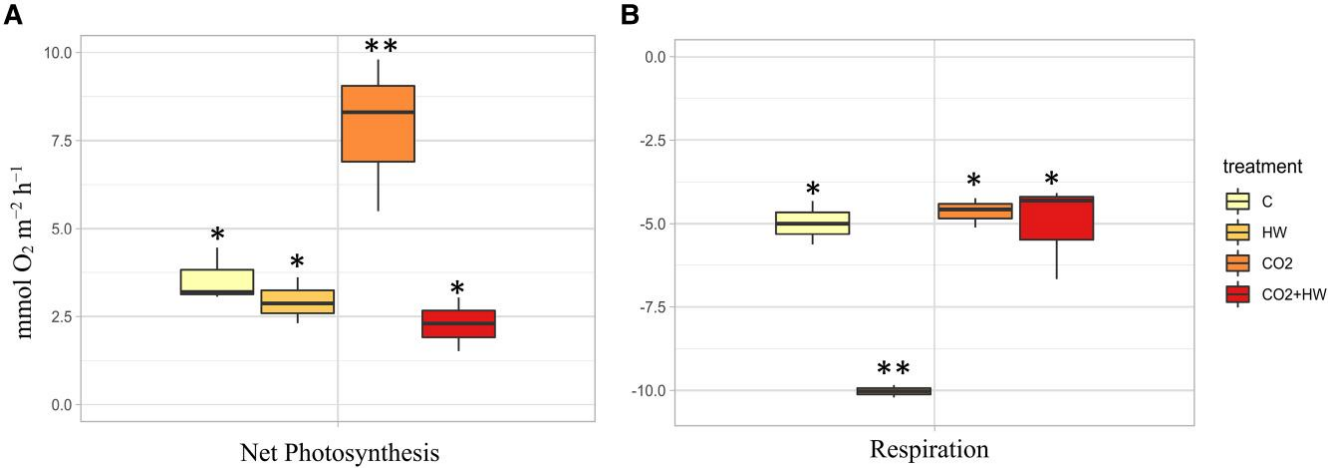
Net photosynthesis (A) and dark respiration (B) measured in seedlings exposed to control (C), high CO_2_ (CO_2_), heatwave (HW), and heatwave under high CO_2_ (CO2+HW) treatments. Asterisks indicate significant differences obtained in the post-hoc Tukey's HSD test.

The maximum photochemical efficiency of PSII (*F*_v_/*F*_m_) was negatively affected by the onset of a simulated marine heatwave (factor HW, *P*<0.01, Supplementary Table S1.1; Supplementary Fig. S3A), while no changes were observed in gross photosynthesis (Supplementary Fig. S3B). In detail, *F*_v_/*F*_m_ decreased in heated seedlings when compared with control (−27.9% in HW and −28.7% in CO_2_+HW) irrespective of the CO_2_ level. The rate of photosynthesis (net photosynthesis, Fig. 2A) doubled under high-CO_2_ (*P*<0.001, HSD post-hoc test), except when combined with heatwave conditions (CO_2_+HW). Dark respiration increased significantly with heatwave conditions, except when in combination with high CO_2_ (*P*<0.001, HSD post-hoc test; Supplementary Table S1.1; Fig. 2B). However, the P:R ratio increased only in high CO_2_, while remaining similar to controls in HW and CO_2_+HW (Supplementary Fig. S3C). The leaf photosynthetic pigment content was not significantly affected by the experimental treatments (Supplementary Fig. S3D).

### Antioxidant response and energy metabolism

**Fig. 3.**
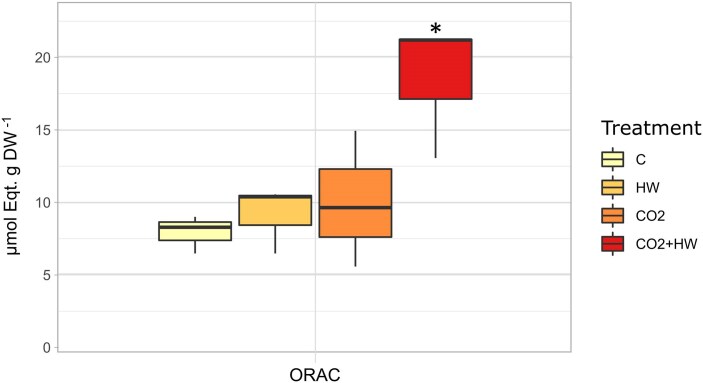
Oxygen radical absorbance capacity (ORAC) measured in control (C), high CO_2_ (CO_2_), heatwave (HW), and heatwave under high CO_2_ (CO_2_+HW) treatments. Asterisk indicates significant differences obtained in the post-hoc Newman–Keuls test.

The ORAC of *P. oceanica* leaves was significantly higher in the CO_2_+HW treatment (Fig. 3; Supplementary Table S1.1), while no changes were detected for HW and CO_2_. The adenylate energy charge (AEC) ratio did not show any significant trends in response to treatments in both leaves and roots (Supplementary Table S1.1). However, a slight increase was observed under CO_2_+HW treatment compared to C in leaves with significant differences observed between leaves and roots (*P*<0.01, three-way ANOVA). TNC measured in leaves and roots across treatments was not affected by treatments (Supplementary Fig. S4B).

### Differentially expressed genes and GO enrichment

**Fig. 4.**
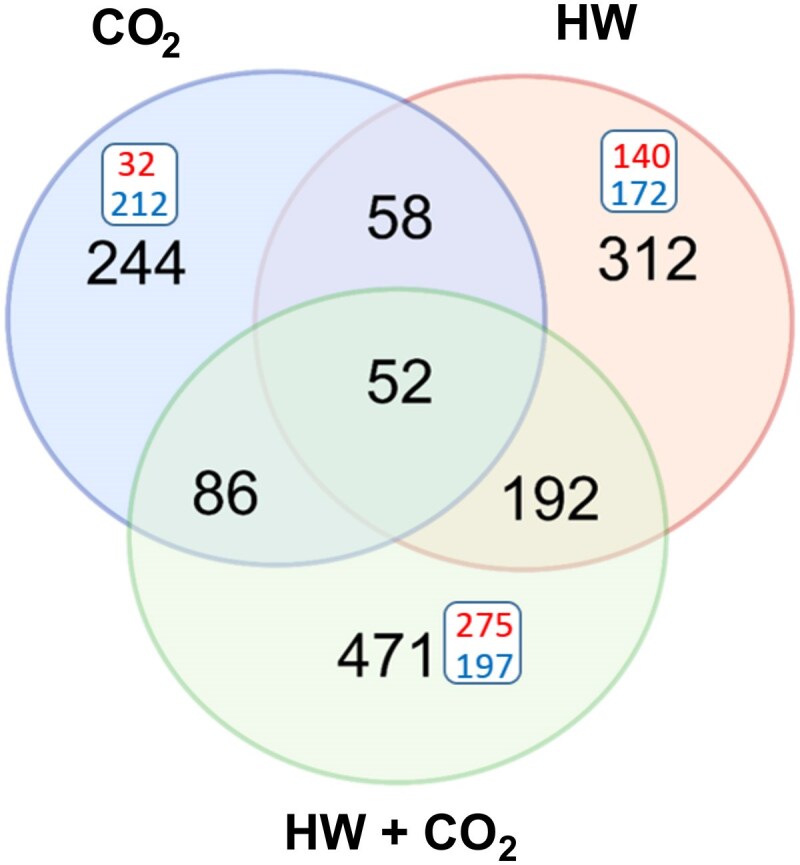
Venn diagrams showing unique and shared number of differentially expressed genes (DEGs) in high CO_2_ (CO_2_), heatwave (HW), and heatwave under high CO_2_ (CO_2_+HW) treatments. The number of repressed (blue) and overexpressed (red) genes are also reported in the squares.

The largest transcriptomic response in a number of DEGs was observed in CO_2_+HW, sharing more DEGs with HW (Fig. 4). Seedlings long exposed to high CO_2_ levels (CO_2_) repressed a large majority of genes rather than over-expressing them. This result contrasts with CO_2_+HW and HW, where the number of up-regulated and down-regulated DEGs was comparable.

#### Heatwave

**Fig. 5.**
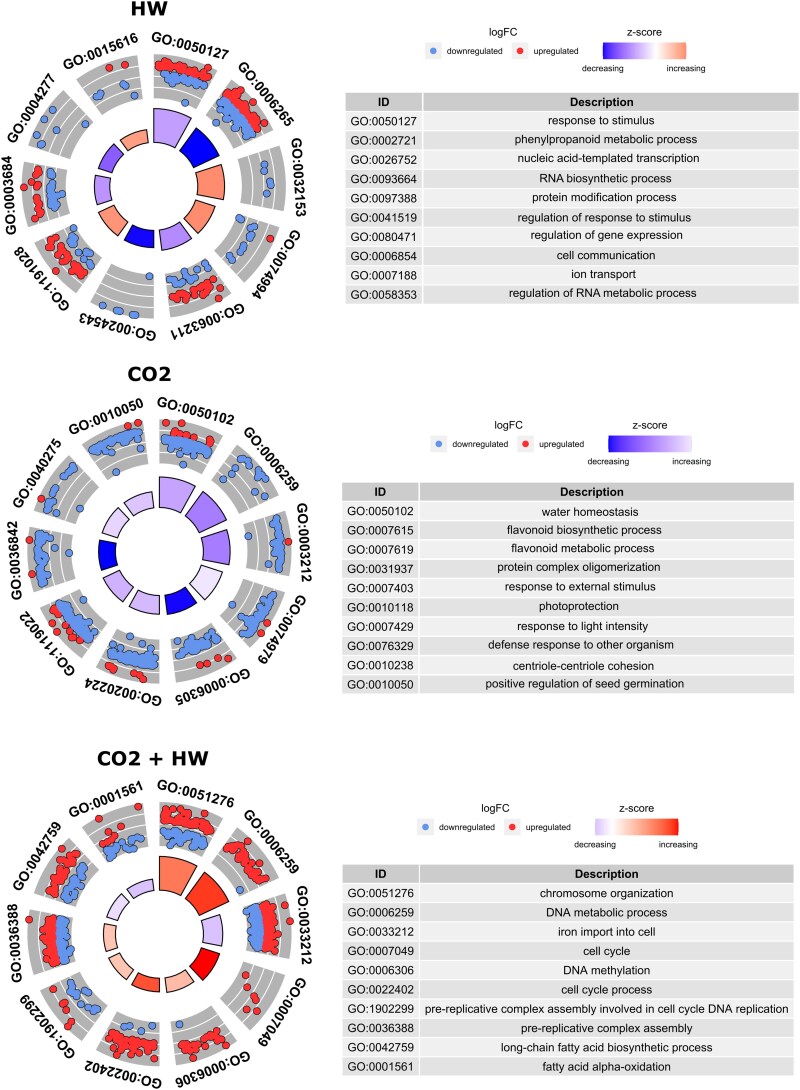
Concentric circle graphs represent differentially expressed genes (DEGs) clustered in the 10 most significant specific biological process terms unique to heatwave (HW) (A), high CO_2_ (CO_2_) (B), and heatwave under high CO_2_ (CO_2_+HW) (C) treatments. Dots refer to up-regulated and down-regulated DEGs. The inner sectors with larger size and darker color represented more significant enrichment.

Heat shock proteins and chaperons were among the most expressed transcripts in HW treatment (18.2 kDa class I heat shock protein, Chaperone protein ClpB1). Plants under HW treatment showed enrichment in specific biological processes related to heat responses (‘pyrimidine-containing compound metabolic process’, ‘response to heat’, and ‘protein folding’; Fig. 5A; Supplementary Tables S2, S3).

#### CO_2_

Plants under CO_2_ treatment showed enrichment in processes involved in flavonoids (‘flavonoid biosynthetic process’ and ‘metabolic process’; Fig. 5B; Supplementary Tables S2, S3) and ‘photoprotection’, where transcripts involved in flavonoid biosynthesis, regulation of cyclic electron flow (CEF) around photosystem I, and photo-protection were down-regulated [*Chalcone synthase* (*CHS*); *Protein proton gradient regulation 5* (*PGR5*); *Photosystem II 22 kDa protein 1* (*PSBS1*)]. Here, vacuolar-sorting receptor and transcripts with oxidoreductase activity were the most expressed (*Vacuolar-sorting receptor 6*, *Cytochrome P450*). Transporters such as *Tonoplast dicarboxylate transporter* (*TDT*) and *ABC transporter G family member 2* (*ABCG2*) were also down-regulated.

#### CO_2_ and heatwave

‘Chromosome organization’, ‘DNA metabolic process’, and ‘DNA methylation’ were exclusively enriched in the CO_2_+HW treatment (Fig. 5C, Supplementary Tables S2 and S3). A key gene involved in the respiratory chain (*AOX1*) was the most up-regulated in CO_2_+HW, but also genes regulating development and cell wall organization [*MIZU-KUSSEI 1* (*MIZ1*) and *Putative pectinesterase 11* (*PME11*)].

### Weighted gene co-expression network analysis

**Fig. 6.**
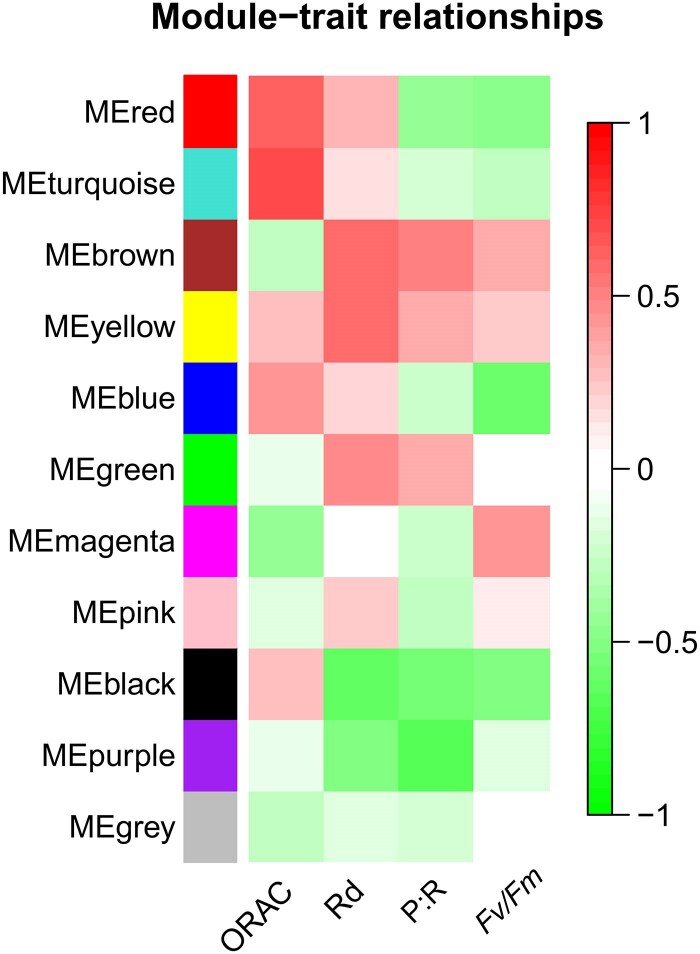
Module–trait relationship of expressed genes significantly correlated with oxygen radical absorbance capacity (ORAC), dark respiration (Rd), ratio of photosynthesis to respiration (P:R ratio), and maximum quantum yield (*F*_v_/*F*_m_) for high CO_2_, heatwave, and heatwave under high CO_2_ treatments. Each row corresponds to a module eigengene (ME) that was defined as the first principal component of each gene module, and the expression of MEs was considered representative of all genes in a given module, a column corresponding to a trait. Each cell contains the corresponding correlation and *P*-value (in brackets). The table is color-coded by correlation according to the color legend.

**Fig. 7.**
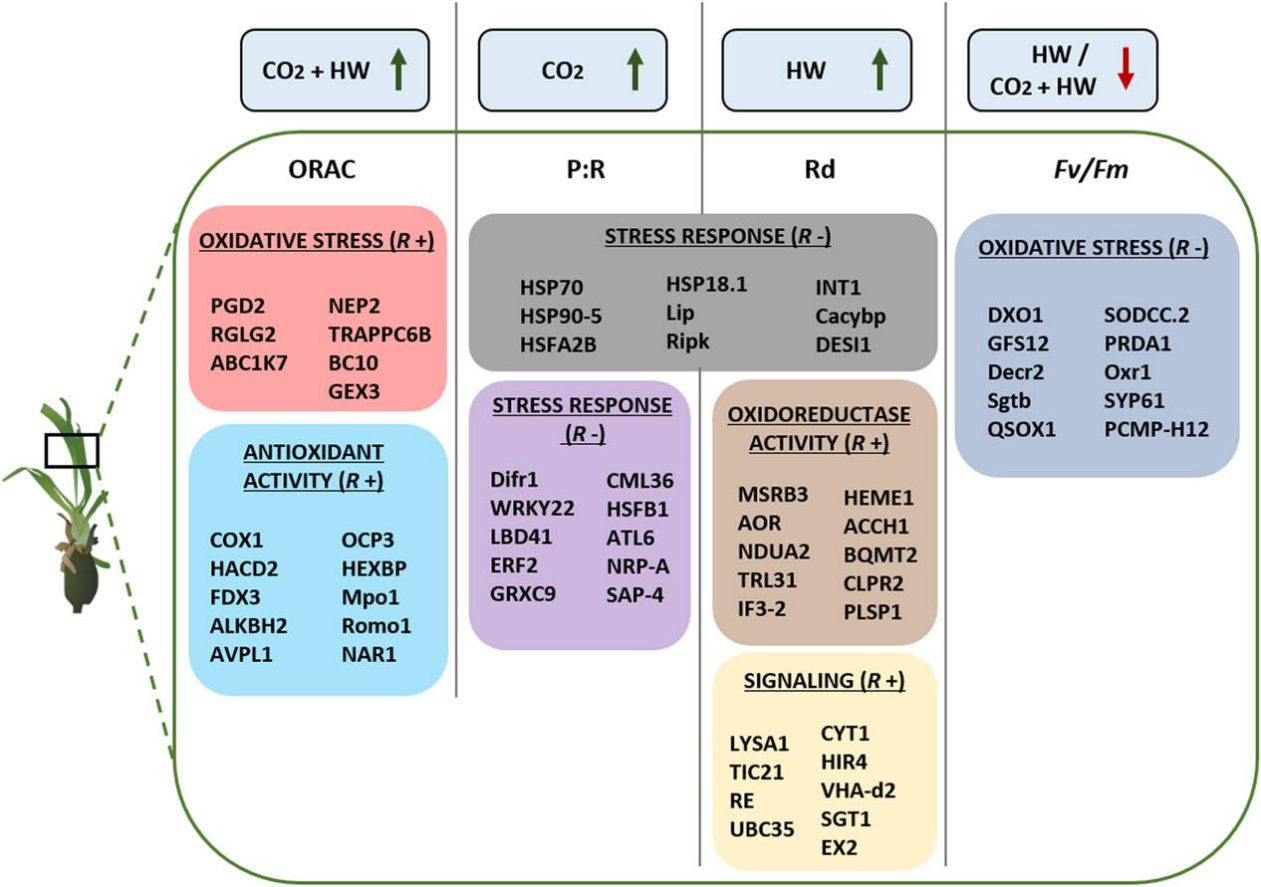
Schematic overview of central genes observed within significant modules correlated with oxygen radical absorbance capacity (ORAC), photosynthesis to respiration ratio (P:R), dark respiration (Rd), and photochemical efficiency (*F*_v_/*F*_m_). Arrows refer to significant results from the analysis of photo-physiological traits (green, increase; red, decrease). Each color corresponds to the corresponding module correlated (*r* +, positive correlations; *r* −, negative correlations) with the photo-physiological trait. Central genes (MM≥0.95) identified in each module are also reported.

WGCNA was performed to investigate genes associated with physiological traits. The WGCNA identified 10 co-expressed gene modules (labeled in different colors in Fig. 6). Among these, six modules (red, turquoise, yellow, blue, black, and purple) were significantly correlated to physiological traits (*R*≥0.6; *P*≤0.05; Fig. 7). Red and turquoise modules were positively correlated with ORAC (MEred, *R*=0.61, *P*=0.03; MEturquoise, *R*=0.72, *P*=0.008). The blue module was negatively correlated with *F*_v_/*F*_m_ (*R*=−0.6, *P*=0.04). Additionally, negative correlations were observed between the black module and dark respiration (Rd; *R*=−0.61, *P*=0.03), as well as between purple and carbon balance (P:R ratio) (*R*=−0.65, *P*=0.02). The yellow module was positively correlated with dark respiration (Rd; *R*=0.6, *P*=0.04).

### Central genes significantly correlated with physiological trait modules

The two major significant modules, including the largest number of co-expressed transcripts, were the blue (*n*=4216 genes) and the turquoise (*n*=4903 genes) modules. Central genes (i.e. hub genes) that have a high degree of connectivity (i.e. MM>0.95) and are related to enriched terms of interest are included in Supplementary Tables S4 and S5.

#### Modules correlated with oxygen radical absorbance capacity

The turquoise module included a total of 1081 central genes (MM>0.95, Supplementary Table S4). Among these, important genes with antioxidant activity were modulated in the CO_2_+HW treatment (*COX11*, *OCP3*, and *FDX3*; Fig. 7; Supplementary Table S6). Central genes were also identified by selecting significant GO-enriched terms within the module. In this case, ‘energy derivation by oxidation of organic compounds’ and ‘carboxylic acid catabolic process’ were enriched in the module. Associated genes were those for important mitochondrial electron transport chain regulators, like cytochromes, NADH dehydrogenases, and alternative oxidases (Supplementary Tables S4 and S5).

The red module included very few central genes (MM>0.95) in comparison with the turquoise one. Several hub genes were implicated in the oxidative and general stress response [*6-Phosphogluconate dehydrogenase decarboxylating 2* (*PGD2*) and *E3 ubiquitin-protein ligase* (*RGLG2*); Supplementary Table S4)], which were particularly modulated in the CO_2_+HW treatment (Fig. 7; Supplementary Table S2). Other genes involved in the ‘detoxification’ and ‘regulation of response to oxidative stress’ were identified (i.e. *U17*, *Glutathione S-transferase F8*, and *Protein activity of bc1 complex kinase 8, chloroplastic* (*ABC1K7*); Supplementary Tables S4 and S5).

#### Modules correlated with respiration and photosynthesis to respiration ratio

In the black module, which was negatively correlated with respiration and P:R ratio, significant genes related to photorespiratory activities were modulated in temperature treatments (HW and CO_2_+HW), including *Glyceraldehyde-3-phosphate dehydrogenase*, *Glutaredoxin-C4*, and *Probable sarcosine oxidase* (Supplementary Table S4). ‘Response to heat’, ‘photomorphogenesis’, and ‘protein folding’ were among the most enriched biological processes. Interestingly, several terms related to the flowering process were also enriched (‘photoperiodism—flowering’, ‘inflorescence development’, and ‘inflorescence meristem growth’). Transcripts included in these terms were particularly modulated under HW treatment (e.g. *Transcription factor GHD7*, *Casein kinase 1-like protein HD16*, and *Protein heading date 3B*). Several transcripts related to mitochondria were also included in this module (*Mitochondrial phosphate carrier protein 1*; *Heat shock protein 90–6, mitochondrial*; *10 kDa chaperonin, mitochondrial*; and *Heat shock 70 kDa protein, mitochondrial*; Supplementary Tables S4 and S5).

The purple module was the least representative one in terms of central genes. Different transcription factors associated with stress response, such as *Zinc finger A20*, *AN1 domain-containing stress-associated protein*, and *Heat stress transcription factor B-1*, were modulated in HW treatment (Fig. 7, Supplementary Table S4). Accordingly, among the most significant enriched terms were ‘responses to stress’ and ‘macromolecule modification’.

Contrary to black and purple modules, the yellow module was positively correlated with only respiration (Figs 6, 7). Among the central genes found in the yellow module, are *Protein EXECUTER 2, chloroplastic* (*EX2*), which is involved in the signaling pathway mediated by singlet oxygen, and *Protein methylene blue sensitivity 1* (*MBS1*), which is required for the acclimation process to ROS. Here, genes involved in plant development were also detected [*Mannose-1-phosphate guanylyltransferase 1* (*CYT1*); *Protein RETICULATA, chloroplastic* (*RE*); Supplementary Table S6]. Structural transcripts forming protein complexes such as mitochondrial membrane ATP synthase (*ATP synthase 6 kDa subunit, mitochondrial* and *ATP synthase subunit O, mitochondrial*), NADH dehydrogenases, and intermembrane chaperones were also found. In addition, several biological processes related to transport activity, apoptotic processes, and protein degradation were enriched (‘nitrogen compound transport’, ‘peptide metabolic process’, ‘regulation of anoikis’, and ‘response to oxidative stress’) (Supplementary Tables S4, S5).

#### Module correlated with photochemical efficiency

The blue module was the only one that negatively correlated with *F*_v_/*F*_m_ (Figs 6, 7). Here, transcripts with antioxidant activity like *Superoxide dismutase [Cu-Zn] 2* (*SODCC.2*), *Oxidation resistance protein 1* (*Oxr1*), and *Peroxisomal 2,4-dienoyl-CoA reductase* were observed. Among enriched terms, different regulatory processes appeared to be modulated, such as ‘DNA repair’, ‘RNA modification’, ‘methylation’, and ‘histone modification’. Some transcripts included in these terms were repressors of the transcription (i.e. *Ethylene-responsive transcription factor ABI4*, *DELLA protein GAI*) (Supplementary Table S2).

### Intersecting differentially expressed genes and weighted gene co-expression network analysis results (key genes in stress responses)

DEGs found for each treatment (HW, CO_2_, CO_2_+HW) overlapping with all transcripts included in significant modules (WGCNA; Supplementary Fig. S5; Supplementary Table S7) were explored to identify key regulators of single and multiple stressors exposure. The results showed that 484 DEGs were related to specific physiological traits (i.e. ORAC, *F*_v_/*F*_m_, Rd, P:R ratio). Among these, 216 DEGs were uniquely regulated in the CO_2_+HW treatment, followed by HW (*n*=186), and CO_2_ (*n*=82). The former included several genes involved in DNA and chromatin modifications such as *ATP-dependent DNA helicase DDM1*, *DNA (cytosine-5)-methyltransferase 1A*, and *Lysine-specific demethylase JMJ25*, which were overexpressed in CO_2_+HW. Other important genes involved in DNA repair mechanisms and oxidative stress response were expressed under CO_2_+HW treatment (*DNA repair protein RAD50*, *Inactive poly [ADP-ribose] polymerase RCD1*). Moreover, the majority of resulting genes were shared between HW and CO_2_+HW. Contrarily, genes with antioxidant activity were repressed in the HW treatment [*Polyphenol oxidase, chloroplastic* (*PPO*) and *Peroxidase 12*]. Here, several heat shock proteins were also expressed (*Heat shock 70 kDa protein 14*, *15*, *81*, *82*, *Heat stress transcription factor A-2b*). Moreover, a key gene involved in the regulation of the circadian clock and auxin pathways (*Reveille 1*) was largely down-regulated in HW, followed by *Polyphenol oxidase, chloroplastic* (*PPO*) and *Glutelin type-D* *1* (*GLUD1*). In the CO_2_ treatment, genes involved in the electron transport [*Photosystem II 22 kDa protein 1, chloroplastic* (*PSBS1*)], cell wall organization [*Probable xyloglucan endotransglucosylase/hydrolase protein 23* (*XTH23*)], and transferase activity [*Leucine-rich repeat receptor protein kinase* (*HPCA1*)] were down-regulated.

## Discussion

In a future scenario of OA, *Posidonia oceanica* will be more capable of coping with the observed and forecasted increase of MHW frequency. Our results, in fact, show that increased levels of CO_2_ in seawater will be a positive factor in enhancing the physiological response of *P. oceanica* seedlings to marine heat waves. Under current CO_2_ concentrations, anomalous high temperatures induced stress on *P. oceanica* seedlings and altered their photosynthetic and respiratory rates, potentially eroding their health and energy status (Figs 2, 3). However, when seedlings were grown under OA conditions, they were able to regulate the transcriptomic machinery to counteract the effects of heat stress by regulating their metabolic rates and enhancing their antioxidant defense (Fig. 5). This is because elevated CO_2_ concentrations, rather than negatively affecting *P. oceanica* seedlings, provide them with greater ‘resources’ in the form of increased carbon accumulation and energy supply that can be used to cope with heat stress conditions, which is reasonable since seagrasses tend to be carbon-limited under natural conditions (Pajusalu *et al*., 2016). Under elevated CO_2_ conditions and no other nutrient limitations, the increased concentration of carbon allows seedlings to enhance their photosynthetic capacity, leading to greater carbon fixation and accumulation, which in turn contributes to withstanding thermal stress. This is the first study conducted on *P. oceanica* seedlings that confirms the beneficial effect of CO_2_ availability in improving seagrass responses to thermal stress. This has been evident in this study, where anomalous warming events cause deleterious effects on *P. oceanica* seedlings (reduction of *F*_v_/*F*_m_, increased respiration rate, and the expression of specific genes), whereas the co-occurrence of acidification of ocean conditions in the coming decades could reverse these negative effects, making the early life stages of seagrasses more resilient to warming than previously thought (Fig. 2). OA could favor recruitment of *P. oceanica* seedlings, whose number is predicted to increase under increased MHW frequency and duration. Their higher sensitivity to temperature increase, in respect to adult plants (Rinaldi *et al*., 2023), creates major doubts about the evolutionary effectiveness of the increased flowering response to global warming.

Under current CO_2_ conditions, *P. oceanica* seedlings exposed to heat showed alterations as already described in heat-stressed seedlings and adults of the species (Marín-Guirao *et al*., 2016; Dattolo *et al*., 2017; Pazzaglia *et al*., 2020, 2022a; Stipcich *et al*., 2022). These alterations include reductions in photosynthetic yields, increases in respiratory metabolic rates at the physiological level, and a strong transcriptomic regulation of heat shock proteins and molecular chaperones at the molecular level. As a consequence of these modifications, the energetic state of plants deteriorates, which in the long term can lead to death (Marín-Guirao *et al*., 2016, 2017; Ruocco *et al.*, 2019). Surprisingly, seedlings that experienced anomalous warming after months of growth in CO_2_-enriched future ocean conditions were able to maintain their respiratory metabolic rate unchanged. Respiratory homeostasis under warming seawater conditions is a functional trait associated with heat tolerance in seagrasses (Collier *et al*., 2011; Marín-Guirao *et al*., 2018), which could be associated with transcriptional regulation at the mitochondrial level (Marín-Guirao *et al*., 2017; Nguyen *et al*., 2021). Seedlings heated under OA conditions indeed show a strong induction of the alternative oxidase (AOX) pathway of the mitochondrial electron transport chain, a response with potential implications for supporting homeostasis in carbon and energy metabolism in stressed plants (Vanlerberghe, 2013). Modifications of the activity of AOX have been described under marine heatwave conditions in *P. oceanica* (Tutar *et al*., 2017; Traboni *et al*., 2018; Ruocco *et al.*, 2019; Pazzaglia *et al*., 2022a) and in terrestrial plants, with implications for leaf respiratory rates (Scafaro *et al*., 2021). In addition to the regulation of cellular respiratory metabolism, one of the main functions of the AOX pathway is the control of ROS production in mitochondria, which increases in plants under conditions of stress and accelerated metabolism (Marquez-Acevedo *et al*., 2023). In our case, seedlings heated in CO_2_-enriched conditions showed an antioxidant capacity (ORAC) twice as high as seedlings in normal CO_2_ conditions (Fig. 3). WGCNA identified transcriptomic modulation involved in this increased antioxidant competence, where ORAC-correlated modules contained a number of core genes involved in the regulation of oxidative stress and antioxidant defense (e.g. *GSTF8*, *FDX3*, *romo1*, *OCP3*) (Figs 6, 7). Therefore, in addition to controlling their respiration rates and ROS production, seedlings grown in future OA conditions were able to activate a higher antioxidant defense and a stronger transcriptomic regulation that was directly involved in the regulation of phenotypic adjustments to cope with anomalous temperatures than seedlings in current pH conditions. This ability could be associated with a better energetic and fitness state acquired while growing under high CO_2_ conditions. Therefore, long-term exposure to a CO_2_-enriched environment has the potential to mitigate the negative effect of abiotic stresses by acting as a fertilizer in several plant species (i.e. a CO_2_ fertilizer effect; Kazemi *et al*., 2018; Shokat *et al*., 2021). Stress mitigation under elevated CO_2_ has already been observed in plants as the result of improved ROS detoxification through the regulation of antioxidants (Ghasemzadeh *et al*., 2010; Zinta *et al*., 2014). Moreover, growth in a CO_2_-rich environment can trigger a priming effect in plants through the activation of metabolic processes and redox signaling, potentially improving their resistance to infections (Mhamdi and Noctor, 2016). This may increase plasticity regulating the response machinery more efficiently in the presence of the additional stressor (Liu *et al*., 2022). Seedlings under these conditions showed much higher photosynthetic rates than seedlings under current CO_2_ conditions, resulting in carbon balances (P:R ratio) twice as high. The mechanism employed by *P. oceanica* seedlings to take advantage of the increased CO_2_ availability appears to be the inactivation of non-photochemical quenching, one of the main photoprotection mechanisms involved in the dynamic regulation of photosynthesis and which has been shown to operate in the species (Marín-Guirao *et al*., 2013). This process is linked to the xanthophyll cycle and requires for its activation the acidification of the thylakoid lumen that occurs under high-light conditions together with the encoding of the PSBS protein (Li *et al*., 2000; Niyogi *et al*., 2005).

In seedlings under OA conditions, the strong down-regulation of the gene encoding the PSBS protein and the PGR5 gene, which contributes to creating a proton gradient across thylakoid membranes (Wang *et al*., 2014), supports this hypothesis. This higher productivity, however, is not reflected in higher carbohydrate content in leaf tissues, possibly because these seedlings were still attached to their seeds. *Posidonia oceanica* seeds contribute to sustaining seedling development until about 1 year after germination, thanks to the large amount of resources they store and their ability to photosynthesize (Balestri *et al*., 2009; Rinaldi *et al*., 2023). It is possible that the sugars produced by seedlings were stored in the seeds since the rhizome, which is the reserve organ of the species, had not yet developed, although the content of non-structural carbohydrates in seeds was not analysed. This possibility is supported by the fact that the biomass of *P. oceanica* seedlings maintained under high CO_2_ concentration for 3 months was almost 2-fold higher than control seedlings but without differences in leaf and root biomass (Hernán *et al*., 2016). Similarly, adult seagrasses can also exhibit an increase in belowground biomass, reflecting increased storage of reserve compounds (Palacios and Zimmerman, 2007). The increased productivity due to months under CO_2_-enriched conditions, with the higher amount of resources stored in the seeds seems to have provided the seedlings with resources to better cope with an anomalous warming event. Moreover, seedling roots were already developed suggesting that reserves were used to build fibers favoring seedling growth. On the other hand, despite their ability to control respiration, heated seedlings under OA conditions showed photosynthetic alterations similar to seedlings heated under the present-day CO_2_ concentration. This is because the photochemical process is impacted by temperature increases, as already described in previous studies (Marín-Guirao *et al*., 2017; Ontoria *et al*., 2019; Nguyen *et al*., 2020). A slight reduction in photochemical efficiency does not significantly affect net photosynthetic rates, probably due to a modification of the activity of alternative O_2_-consuming metabolic pathways (photorespiration, the Mehler reaction, cellular respiration, chlororespiration, and the mitochondrial alternative oxidase pathway) as described for this and other seagrass species subjected to abiotic stress, including heat, salinity, or high light (Touchette and Burkholder, 2000; Silva *et al*., 2013). However, the prolonged exposure to elevated CO_2_ equipped the seedlings to better cope with stress by activating specific regulatory pathways (i.e. antioxidants, photoprotection), thereby maintaining a stable carbon balance. Noteworthy, genes involved in DNA and chromatin modifications like *DDM1* and *JMJ25* were overexpressed in seedling grown under increased CO_2_ concentration. Their expression patterns correlated with antioxidant capacity and photochemical efficiency, respectively. Besides DNA methylation being poorly investigated in *P. oceanica*, recent evidence has described a key regulatory and dynamic role of the process in response to heat stress (Pazzaglia *et al*., 2023). In particular, *DDM1* encodes DNA (cytosine-5)-methyltransferase 1A, which is a Snf2 remodeler essential for normal DNA methylation in plants (Zemach *et al*., 2013). Recently, the increase in DNA methylation levels was described during seed development in plants, and potentially related to the antioxidant capacity under temperature changes (Cui *et al*., 2022). A similar relationship was also observed for Lysine-specific demethylase JMJ25, which is implicated in the regulation of anthocyanin biosynthetic genes. This group of flavonoids is involved in the response to abiotic stress by scavenging ROS and reducing oxidative stress. These results opened further questions on the potential regulatory role played by DNA methylation in mediating stress responses in seagrasses.

In conclusion, this study revealed that the future increased availability of CO_2_ in the oceans could foster productivity of *Posidonia oceanica* meadows, making young plants more resilient to global environmental stressors and local human-derived threats and better equipped for developing into adult plants. Further multiple-stressor assessments in different seagrass species are needed, considering the effects of OA on the whole seagrass ecosystem including the associated communities. Although our study did not include sampling before CO₂ treatment or between the CO₂ and heatwave phases, future studies with time-series sampling could better track metabolic changes providing a more comprehensive understanding of the acclimation process and stress responses. Additionally, integrating more ecologically realistic simulations—such as natural patterns of temperature fluctuation and variable stress exposure across time—would enhance our ability to predict long-term plant performance under future climate scenarios. Nevertheless, considering the paramount importance of ecosystem services provided by seagrass meadows and their role in enhancing human health and human livelihood along the coastline, these results represent a positive message and a potential ray of light for the future.

## Supplementary Material

eraf276_Supplementary_Data

## Data Availability

Raw reads are freely available at the Sequence Read Archive (SRA) partition of NCBI database (PRJNA577416, reviewer link: https://www.ncbi.nlm.nih.gov/bioproject/PRJNA1131296).

## Supplementary data

The following supplementary data are available at *JXB*  online.

Fig. S1. Photosynthetic performance (*F*_v_*F*_m_) during the experimental phase.

Fig. S2. Scale independence and mean connectivity analysis.

Fig. S3. Photosynthetic performance metrics in seedlings under treatments.

Fig. S4. Metabolic indicators in leaves and roots under treatments.

Fig. S5. Number of genes overlapped with weighted co-expression analysis (WGCNA) and differentially expressed genes (DEGs).

Table S1. Two-way ANOVA for the photo-physiological and biochemical traits measured in seedling treatments.

Table S2. Differentially expressed genes (DEGs) across treatments.

Table S3. GO enrichment analysis performed for seedlings exposed to treatments.

Table S4. Transcripts included in the most significant modules.

Table S5. GO enrichment in significant modules.

Table S6. Hub genes selected from significant modules.

Table S7. Transcripts that were both differentially expressed (DEG) and significantly correlated with plant traits.
