# Supplementary material for: Handling the heat: ocean acidification mitigates the effects of marine heatwaves on Posidonia oceanica seedlings
Source: J Exp Bot. 2025 Jun 24;76(22):6958–73. doi: 10.1093/jxb/eraf276 (PMC12675272; doi:10.1093/jxb/eraf276)
Supplement: eraf276_Supplementary_Data [file eraf276_supplementary_data.zip › jexbot314940-file001.pdf]

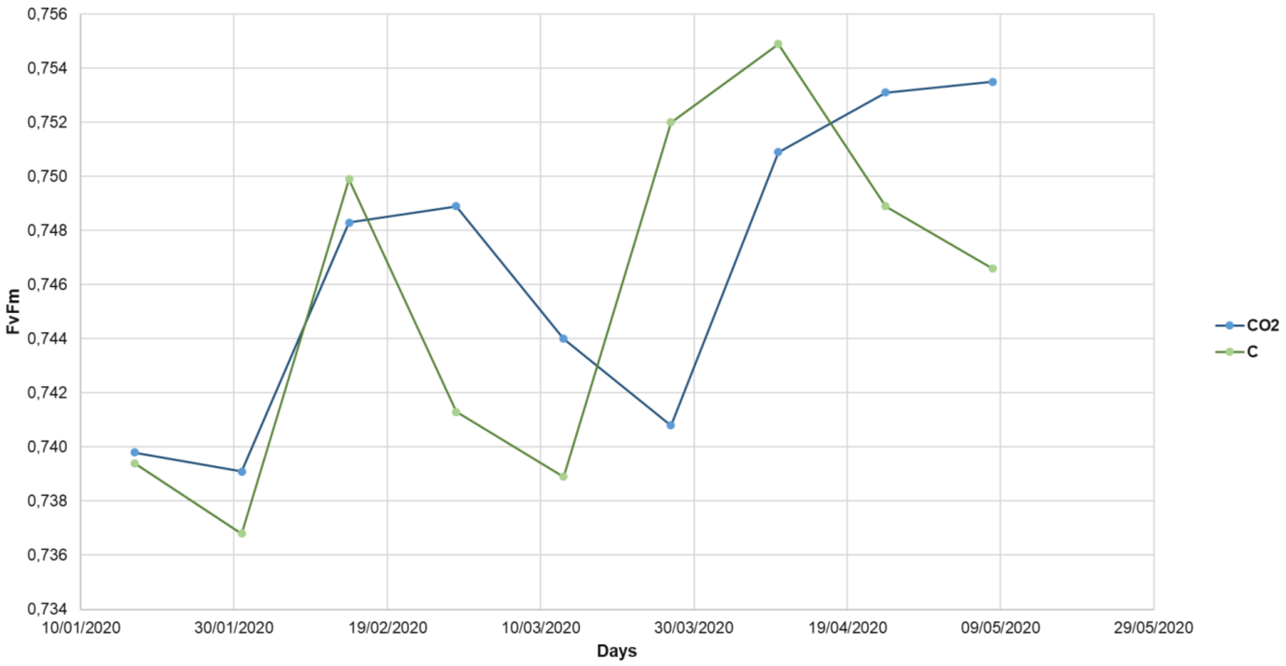

Figure S1. Maximum quantum yield (Fv/Fm) measured in seedlingsgrown unde high CO_2 levels (CO2) and control conditions during the experimental phase.

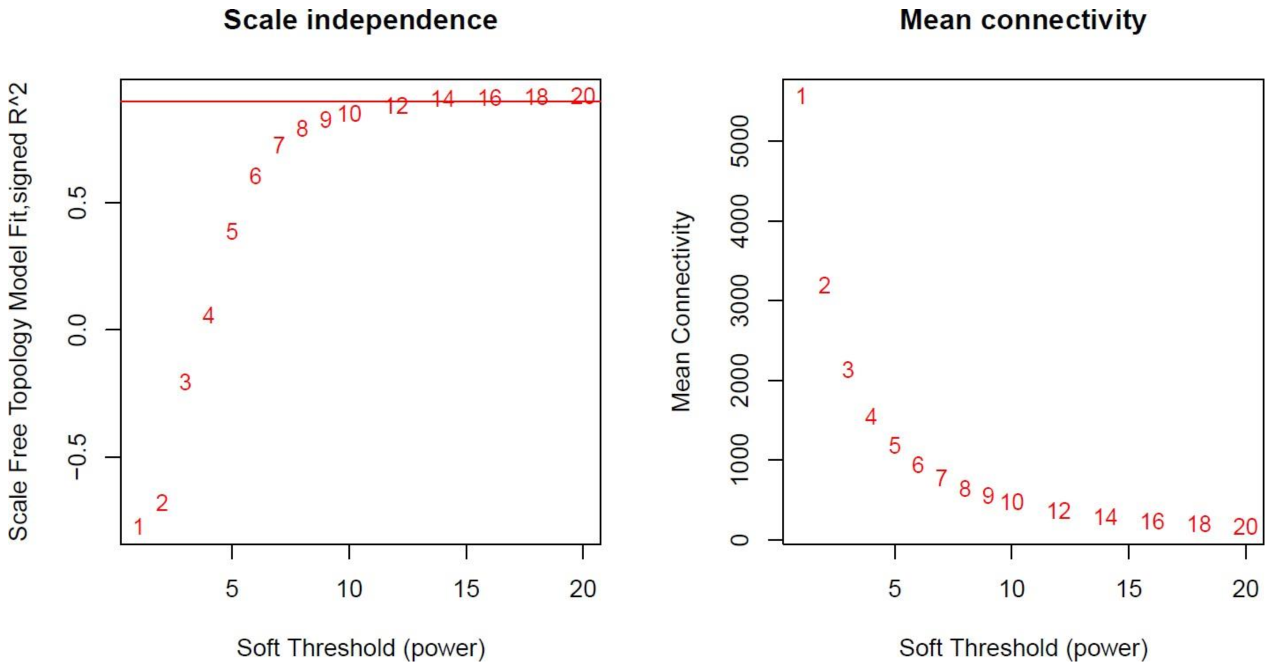

Fig. S2 Scale independence and mean connectivity analysis.

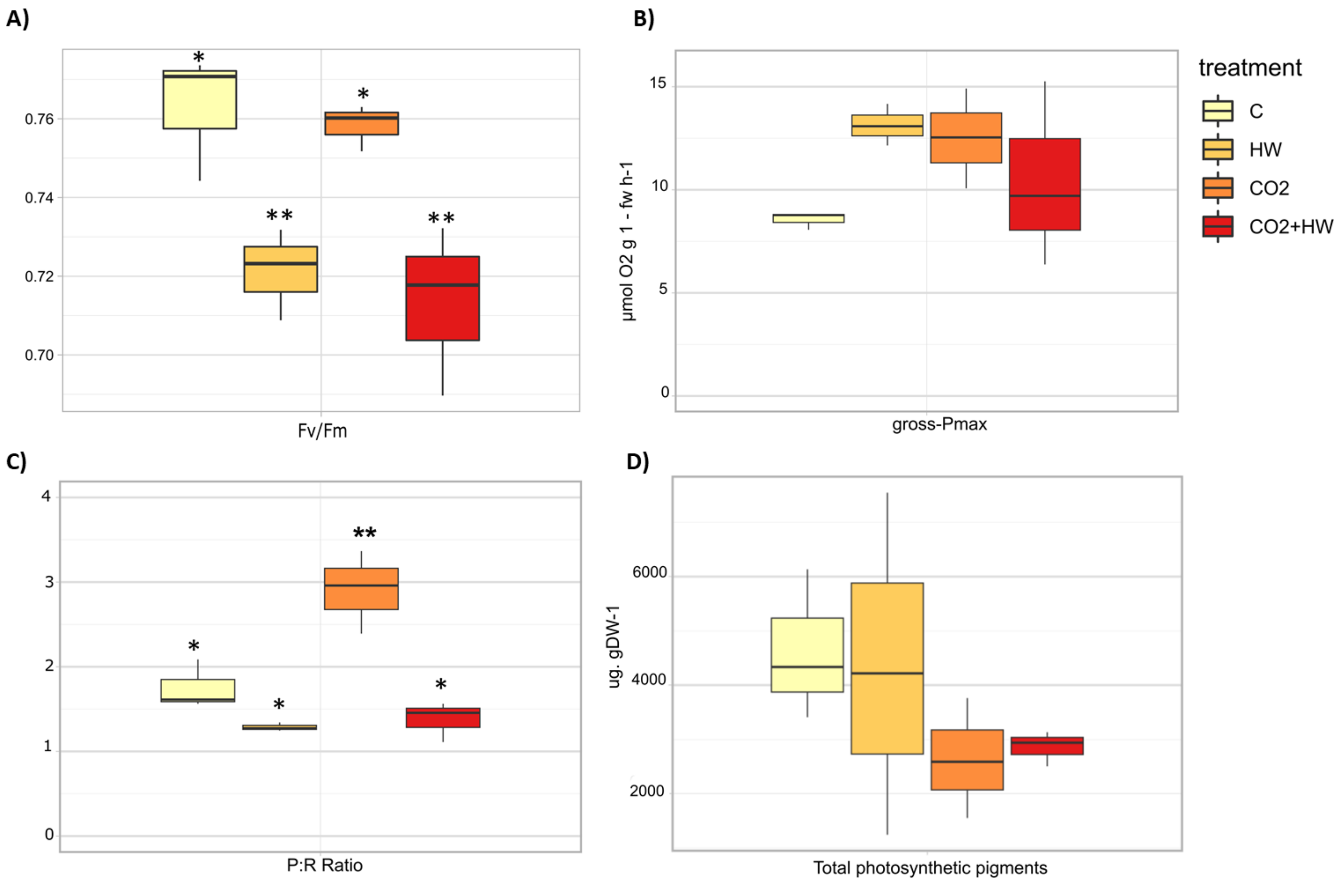

Figure S3. Maximum quantum yield (Fv/Fm, **A**), gross-photosynthesis (gross-Pmax, **B**) and gross-photosynthesis: dark-respiration ratio (P:R Ratio, **C**), and Total photosynthetic pigments (**D**) measured in control (C), seedlings exposed Control (C); High CO2 (CO_2); Heatwave (HW), Heatwave under high CO_2 (CO_2HW) treatments. Asterisks indicate significant differences obtained in the post hoc Tukey's HSD (honestly significant difference) test.

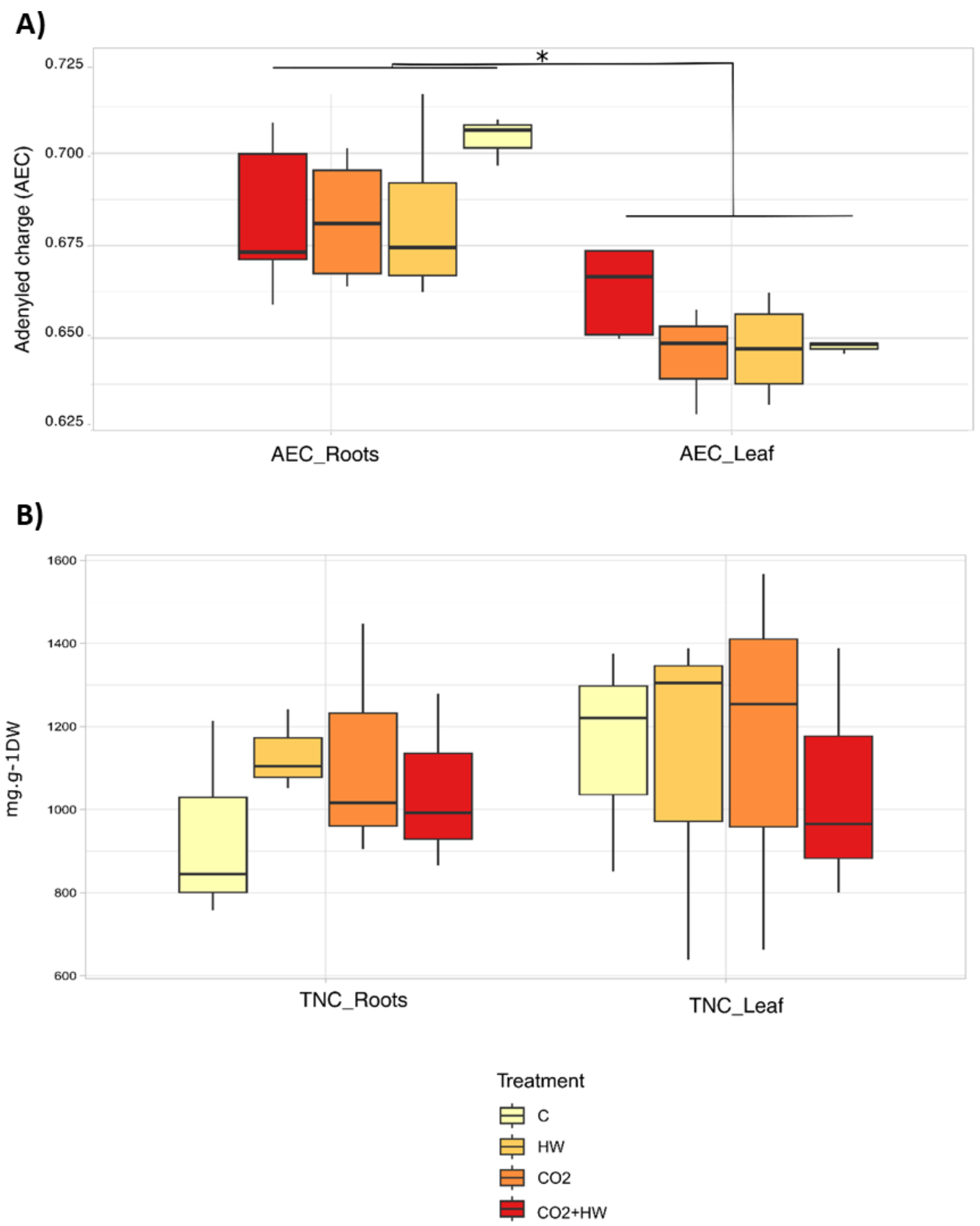

Figure S4. Adenylate Energy Charge (AEC, **A**) and Total non-structural carbohydrates (TNC, **B**) measured in the leaf and roots exposed to Control (C); High CO_2 (CO_2); Heatwave (HW), Heatwave under high CO_2 (CO2+HW) treatments. Asterisks indicate significant differences obtained in the post hoc Tukey's HSD (honestly significant difference) test.

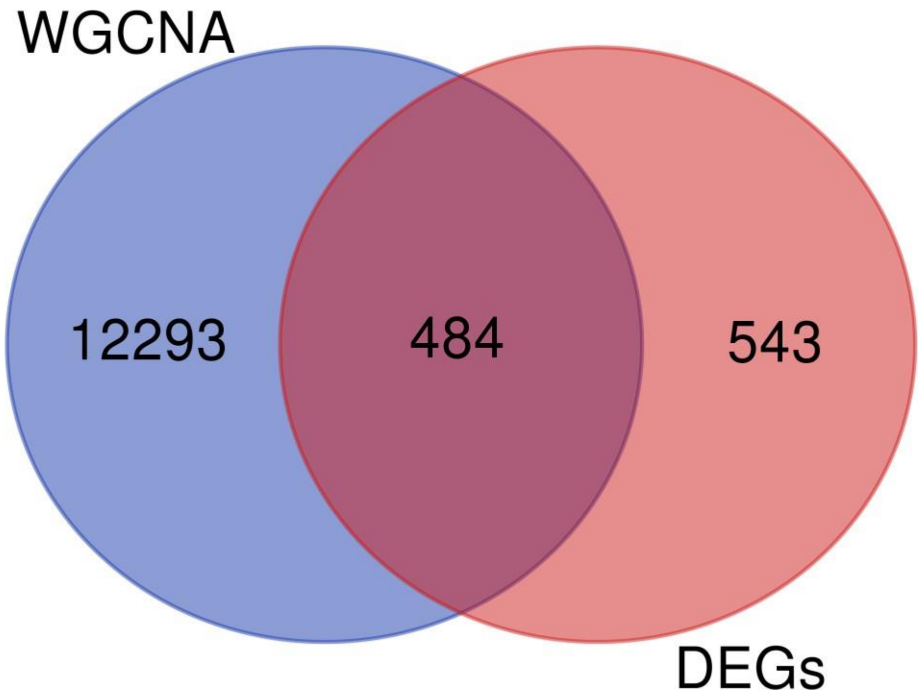

Figure S5. The Venn diagram representing the number of genes selected by the significant modules in the weighted co-expression analysis (WGCNA) overlapped with the number of differentially expressed genes (DEGs) found across all treatments.
