# Supplementary material for: Handling the heat: ocean acidification mitigates the effects of marine heatwaves on Posidonia oceanica seedlings
Source: J Exp Bot. 2025 Jun 24;76(22):6958–73. doi: 10.1093/jxb/eraf276 (PMC12675272; doi:10.1093/jxb/eraf276)
Supplement: eraf276_Supplementary_Data [file eraf276_supplementary_data.zip › jexbot314940-file003.docx]

**Handling the heat: Ocean acidification mitigates the effects of marine heatwaves on *Posidonia oceanica* seedlings**

**Jessica Pazzaglia^1,2^, Làzaro Marin-Guirao^3^, Luca Ambrosino^4^, Katia Pes^5^, Monya Costa^6^, Isabel Barrote^7,8^, João Silva^8^, Gabriele Procaccini^1,2^**

^1^Department of Integrative Marine Ecology, Stazione Zoologica Anton Dohrn, Villa Comunale, 80121 Naples, Italy

^2^National Biodiversity Future Center, Palermo, Italy

^3^Centro Oceanográfico de Murcia, Instituto Español de Oceanografía (IEO, CSIC), 30740 San Pedro del Pinatar, Murcia, Spain

^4^Department of Research Infrastructure for Marine Biological Resources, Stazione Zoologica Anton Dohrn, Villa Comunale, 80121, Napoli, Italy

^5^Excellence Cluster Cardio-Pulmonary Institute (CPI), Justus-Liebig-University, Giessen, Germany

^6^Greencolab- Associação Oceano Verde, Universidade do Algarve, 8005-139 Faro, Portugal

^7^Faculdade de Ciências e Tecnologia, Campus de Gambelas, Universidade do Algarve, 8005-139 Faro, Portugal

^8^Centre of Marine Sciences (CCMAR/CIMAR LA), Campus de Gambelas, Universidade do Algarve, 8005-139 Faro, Portugal

**ORCIDs**

**Jessica Pazzaglia: https://orcid.org/0000-0002-8677-7712**

**Luca Ambrosino: https://orcid.org/0000-0003-1756-9538**

## **Isabel Barrote: https://orcid.org/ 0000-0002-0922-0259**

**Monya Costa: https://orcid.org/ 0000-0002-4236-9063**

**Làzaro Marin-Guirao: https://orcid.org/0000-0001-6240-8018**

**João Silva: https://orcid.org/**[**0000-0002-7211-1661**](https://orcid.org/0000-0002-7211-1661)

**Gabriele Procaccini:** [**https://orcid.org/0000-0002-6179-468X**](https://orcid.org/0000-0002-6179-468X)

**Highlight statement**

Ocean acidification enhances *Posidonia oceanica* seedling resilience to marine heatwaves by modulating antioxidant defenses, metabolism, and gene expression, potentially mitigating climate change impacts on seagrass ecosystems.

**Summary**

Ocean acidification (OA) and marine heatwaves (MHWs) are key drivers of marine ecosystem changes that can interact and influence marine organisms. Seagrasses, including the long-lived *Posidonia oceanica* endemic to the Mediterranean Sea, are widely distributed along coastal habitats, forming highly valuable underwater meadows. The germination and survival of the early life stages of *P. oceanica* are strongly affected by environmental changes. To assess the impact of warming and acidification on its future, we conducted a multifactorial experiment where *P. oceanica* seedlings were grown under OA conditions for six months and then exposed to a seawater warming event. Seedlings’ performance was investigated by analyzing photo-physiology, antioxidant capacity, energetic metabolism and transcriptomic profiles. The Weighted Gene Correlation Network Analysis (WGCNA) was used to integrate phenotypic plant traits with transcriptomic results to identify central genes involved in plant responses to OA and temperature exposure. Results demonstrated that prolonged OA exposure enhances *P. oceanica* seedling resilience to MHW. Specifically, seedlings regulated their antioxidant systems and transcriptomic machinery to better cope with thermal stress. Under current CO_2_ concentrations, elevated temperatures induced stress in *P. oceanica* seedlings, impacting photosynthesis and respiration. However, OA could mitigate the impact of warming in the future, enhancing *P. oceanica*'s resilience to global stressors.

**Keywords:** *seagrasses, multiple stressors, heatwave, acidification, physiology, energetic metabolism, antioxidant response, transcriptomics, acclimation*

**1. Introduction**

Carbon emissions from human activities are threatening coastal marine ecosystems worldwide. Over the last 250 years, oceans have become a carbon dioxide (CO_2_) sink, uptaking between 20–30% of total emissions of atmospheric CO_2_ (Bindoff et al., 2019). The projected time of emergence placed ocean acidification (OA) as the primary driver of marine ecosystem changes, followed by ocean warming, which is also correlated with the increase of CO_2_ in the atmosphere (IPCC Climate Change, 2013). Longer and more intense anomalous events of seawater warming (i.e., marine heat waves, MHWs) are more frequent, and their duration globally increased by 54% during the last decades (annual MHW days, Oliver *et al*, 2018). Considering also the emerging local stressors in coastal habitats (e.g. eutrophication), 97.7% of the oceans are globally affected by more than one stressor that potentially interacts with others, exacerbating the overall impact on individuals, populations, and ecosystems (Halpern *et al.*, 2015). The seagrass species *Posidonia oceanica* is endemic to the Mediterranean Sea, where it grows along the coasts of the whole basin, except for the southeastern region, showing a clear genetic structure among populations distributed along bathymetrical and latitudinal gradients (Jahnke *et al.*, 2019; Procaccini *et al.*, 2023). *P. oceanica* is a key foundation species hosting 25% of the total biodiversity of the Mediterranean basin (Relini 2008), storing 14.6–36.9 million t C_org_ in the matte system, (Monnier *et al.*, 2022) regulating biogeochemical cycles and protecting coasts from erosion (Ondiviela *et al.*, 2014). Distinct populations also show differential transcriptomic responses when exposed to simulated sea warming experiments (Marín-Guirao *et al.*, 2017; Marín‐Guirao *et al.*, 2019; Pazzaglia *et al.*, 2022*b*). Extensive *P. oceanica* meadows can counteract possible adverse effects of acidification by modifying the pH in the water column (Hendriks *et al.*, 2014) while sequestering and immobilizing carbon in their rhizomes.

Seagrasses are expected to benefit from high levels of CO_2_ in seawater, increasing carbon fixation and thus photosynthesis (Koch *et al.*, 2013), simultaneously reducing photorespiration. In the context of climate change, rising temperature alters the carbon balance in the plant (Nguyen *et al.*, 2021) and promotes the accumulation of reactive oxygen species (ROS) (Tutar *et al.*, 2017). Although the isolated effects of OA and temperature on seagrass performance have been explored considering photo-physiological responses (Apostolaki et al., 2014; Deguette et al., 2022; Pedersen et al., 2016; Piro et al., 2020; Rubio et al., 2020), energetic metabolism (Beca-Carretero *et al.*, 2018; Pacella *et al.*, 2018), epiphyte communities (Cox *et al.*, 2015; Ramajo *et al.*, 2019; Lee *et al.*, 2022), and gene expression (Olivé *et al.*, 2017; Ruocco *et al.*, 2017), there are only a few studies reporting their combined effects (*Zostera marina*, Repolho *et al*, 2017, Zayas-Santiago *et al.*, 2020; *Thalassia hemprichii* Listiawati & Kurihara, 2021; *Cymodocea serrulata,* Viana *et al*, 2023). Several studies have revealed inter- and intraspecific responses of adult seagrass plants to various stressors (York *et al.*, 2013; Dattolo *et al.*, 2014; Ontoria *et al.*, 2019; Guerrero-Meseguer *et al.*, 2020*a*; Pazzaglia *et al.*, 2020, 2022*b*; Ruocco *et al.*, 2020; Kaldy *et al.*, 2022), while little is known about the effect of multiple stressors on the early life stages (*P. oceanica*, Guerrero-Meseguer, *et al*, 2020, 2017; Pereda-Briones *et al*, 2019; Hernán *et al*, 2017; Rinaldi et al 2023). For instance, only Lowell *et al*, (2021) and Hernán *et al*, (2016) investigated the OA effect on *Z. marina* seedlings, and no one has studied the impact of both long-term OA and marine heat wave (MHW) exposure.

Seagrass genotypes have large phenotypic plastic properties that have favored species diversification and the colonization of heterogeneous environments (Pazzaglia *et al.*, 2021; Sandoval-Gil *et al.*, 2023). This is the case of *P. oceanica,* which occurs from 1m to 40m in depth and colonizes heterogeneous environments where environmental features (i.e. salinity, turbidity, temperature, and light conditions) show clear gradients (Procaccini *et al.*, 2017). Seagrasses also have different colonization strategies, ranging from short-lived plants with small seeds that germinate close to the parent meadows (*Halophyla* sp*.*, Gu *et al*, 2022), to long-living ones that form buoyant fruits with large dispersal capacity (*Posidonia* sp., Guerrero-Meseguer *et al*, 2018). In the context of climate change, seagrasses can respond in different ways to unfavorable conditions (Nguyen *et al.*, 2021). For instance, flowering events observed in *P. oceanica* meadows were positively correlated with mean summer sea surface temperature (SST) (Stipcich *et al.*, 2024), and were identified as a stress response with potential adaptive consequences for the species (Ruiz *et al.*, 2018; Marín-Guirao *et al.*, 2019). Nevertheless, little is known about the capability of seedlings to establish and grow in a future environmental scenario, under increased OA and MHWs frequency.

Thus, exploring mechanisms that favor seagrass adjustments to stressful conditions (i.e., acclimation) is important to understand their biology and predict future responses to environmental changes. Most studies performed on *P. oceanica* seedlings have analyzed stress responses at morphological, physiological, and biochemical levels (Hernán *et al.*, 2016; Guerrero-Meseguer *et al.*, 2017; Pazzaglia *et al.*, 2022*a*; Stipcich *et al.*, 2022*a*), while whole-genome transcription analysis is completely unexplored (but see, Pazzaglia, et al., 2022). While photo-physiological and metabolic analyses are valuable tools for assessing plants' performance in response to stress exposure, molecular approaches, such as RNA sequencing (RNA-seq) and DNA methylation (DNAm), can quantitatively analyze changes in gene expression and methylation at a specific time point and state, revealing complex regulatory networks (Anderson and Mitchell-Olds, 2011). It is widely known that multilevel crosstalk mechanisms based on gene transcripts and epigenetics regulate phenotypic changes in the presence of environmental shifts, forming a complex machine that promotes fast reactions and adjustments to newly imposed conditions (Pazzaglia *et al*, 2021; Hansen *et al*, 2022; Xi *et al*, 2020). Under stressful conditions, genes cooperate and are modulated in clusters (e.g. co-expression) to activate regulatory strategies (Williams and Bowles, 2004). Co-expressed gene clusters can be functionally similar or have common biological regulatory roles. Novel bioinformatics applications, like the weighted gene co-expression network analysis (WGCNA), build correlation networks of clustered genes (i.e., modules) that are represented by module eigengene (ME) values and correlate modules to phenotypic traits (Langfelder and Horvath, 2008). The power of this analysis is to identify a cluster of genes differentially expressed and significantly involved in regulating a phenotypic trait under stress exposure (Yu *et al.*, 2020). In plants, the integration of phenotypic responses with transcriptomic has been a useful approach for identifying pools of genes directly involved in the regulation of the stress response (Amrine *et al.*, 2015; Reimer *et al.*, 2021; Riccio-Rengifo *et al.*, 2021; Ju *et al.*, 2023). A similar analysis was recently applied also in *P. oceanica* adult plants to search for early warning indicators of stress signals derived from temperature and nutrient stressors (Santillán-Sarmiento *et al.*, 2023). In that case, shoot survival was integrated with transcriptomic profiles through the WGCNA allowing the identification of several transcripts involved in stress-related biological processes.

In the present study, we aimed to explore how prolonged exposure to OA affects the ability of *P. oceanica* seedlings to cope with MHW, simulating a predicted future scenario of OA and MHW co-occurrence. For this purpose, we performed a multifactorial experiment where *P. oceanica* seedlings were grown under OA conditions for six months and then exposed to an anomalous seawater warming event. Phenotypic traits such as photo-physiology, antioxidant capacity, and energetic metabolism were analyzed and integrated with transcriptomic data by applying the WGCNA to explore potential interactive effects between OA conditions and temperature increase.

**2. Materials and Methods**

**2.1 Experimental design and seedlings collection**

*P. oceanica* seeds collected in June 2019 along the coasts of Marsala (West Sicily) were left to germinate and grow as described in Pazzaglia *et al*. (2022). By the end of October, seedlings were shipped by plane in thermic flasks to the Centre of Marine Sciences (CCMAR) in Faro, Portugal, where they were immediately transplanted in individual small seed pots (5 × 5 × 6 cm) filled with marbles and allocated randomly in twenty fully independent 20L experimental aquaria (15 seedlings per aquarium) (**Fig. 1B**), installed in an indoor mesocosm facility. The system was an open-circuit type, with each aquarium being fitted with an aeration unit and a small water circulation pump (**Fig. 1C)**. Seawater was pumped from a coastal lagoon (Rio Formosa) adjacent to CCMAR marine station and pre-filtered (sand filters, in-line cartridge filters and a 16W UV filter), before entering the mesocosm facility. Long-term monitoring data from the coastal lagoon indicate relatively stable conditions in term of salinity and alkalinity that consist of 34 - 37 ppt and 2450 - 2600 μmol kg⁻¹, respectively. At the beginning of the acclimation period, the photoperiod was set to 10:14 (light: dark, hr). The photoperiod was changed periodically during the experiment following the natural number of light: dark hours.

After six weeks of acclimation to mesocosm conditions under normal pCO_2_, ambient water temperature, and 55.6 ± 3.3 μmol photons/m^2^/s of light intensity, pCO_2_ was gradually increased in half of the tanks until a maximum of 750-900 ppm (water pH 7.790± 0.062) (**Fig. 1A**). CO_2_-enriched air was continuously prepared in a large‐volume premix tank (4,000 L) where industrial grade CO_2_ was blended with air to obtain the target pCO_2_. The premix tank was controlled via direct analysis of CO_2_ using an infrared gas analyzer (IRGA) (WMA‐4, PPSystems) coupled to a PID controller (PID330; TEMPATRON) that operated a solenoid valve to regulate the CO_2_ flush into the premix tank. The CO_2_-enriched air was pressure-pumped into a distribution pipe and readily available on tap in the mesocosm facility. In the tanks assigned with the high-CO_2_ treatment this enriched blend was bubbled into the individual aeration units, from where CO2-enriched water circulated through the aquarium. The effectiveness of the system in attaining the desired CO_2_ levels and a proper homogenization of the conditions throughout the aquarium were thoroughly tested beforehand. The remaining tanks were kept under ambient temperature and pCO_2_ (water pH 8.186±0.050 ). Seedlings were grown under high CO_2_ and control conditions for six months (until mid-June).

In June, the water temperature in the aquaria was set and stabilized at 24°C, reflecting the average temperature of the water entering the system during the proceeding month. After 1 week at 24°C, a heatwave was simulated in half of the high CO_2_ aquaria (five aquaria) and half of the control CO_2_ aquaria (five aquaria) by gradually increasing temperature along a heating ramp (1°C /day) up to 32°C. Throughout the entire acclimation and experimental period, the aquaria's water temperature and pH were monitored daily using HOBO Pendant data loggers (Onset Corp.) and a Thermo Scientific Orion 8103SC pH meter, respectively. Water temperature was additionally monitored daily (Roth digital thermometer, Hanna) in each aquarium at the warmest time of the day (between 2 and 4 p.m.). Irradiance was measured periodically in every tank (LI-192+LI-250, Licor). pCO2 was monitored continuously with a non-dispersive Infrared Gas analyzer (WMA-4, PP Systems, UK) coupled to a gas exchange column (Mini-Module membrane contactor, Celgard, USA). Salinity (CO310 conductivity meter, VWR) was also measured daily, between 11 a.m. and 1 p.m., in all tanks.

After five days at 32°C, leaf samples were collected from the seedlings under the different experimental conditions (n=5): Control (C, T=24°C, current CO_2_); High CO_2_ (CO_2,_ T=24°C, 700ppm CO_2_); Heatwave (HW, T=32°C, ambient CO_2_); Heatwave under high CO_2_ (CO_2_HW, T=32°C, 700 ppm CO_2_). Leaves were cleaned of epiphytes, rinsed with distilled water, blotted dry, and stored at ‑80°C until analysis for photosynthetic pigments, antioxidant capacity, adenylate charge, soluble sugars, and starch content. The youngest leaves (2- and 3-ranked leaves) were collected per each treatment for transcriptomic analysis, gently cleaned of epiphytes, and entirely submerged in RNAlater© (Ambion, life technologies).

**2.2 Photo-physiological assessment**

The potential quantum yield of photosystem II (Fv/Fm) was measured avery two weeks during the experimental periods (**Fig. S1**) in one plant per tank at the middle section of the youngest or second-youngest leaf using a pulse amplitude-modulated fluorometer (Diving-PAM, Heinz Walz, Effeltrich, Germany). Fv/Fm was measured, after the dark period and before switching on the lights. Photosynthesis-Irradiance curves (PI-curves) were measured in *P. oceanica* leaves of all the aquaria at the end of the heatwave (n=5). The setup comprised five independent chambers filled with water from the aquaria and sealed with a Petri dish containing an optical O_2_ sensor (Presens Spot PS). The water conditions inside each incubation chambers, was kept at the same temperature and pH as in the aquaria by a closed-circuit thermostatic water-bath temperature controller (Julabo HC, Julabo Labortechnik, Seelbach, Germany); magnetic stirrers ensured water homogenization inside the incubation chambers. Five LED lamps provided the light, and the 9 different light intensities plus the dark were achieved by using various combinations of neutral-density filters. Leaf samples (n=5) were collected, cleaned from epiphytes, and immediately placed inside the incubation chambers in the dark. After a 20-minute dark acclimation period, O_2_ concentration (μmol/L) was measured in each chamber with a Microx 4 PreSens Optode (Regensburg, Germany). Then, a further 15-minute dark incubation was done to assess dark respiration. The light was then switched on, and after a 15-minute incubation under the lower light intensity, O_2_ concentration (μmol/L) was measured in each chamber. The same procedure was followed for increasing light intensities of photosynthetically active radiation (PAR) within the range of 0 to 1252 μmol/L photons/m^2^/s. Net photosynthetic (NP) and dark respiration (DR) rates were calculated as follows:

(([O_2_]_f_ – [O_2_]_i_) xV)/T/A

Where [O_2_]_f_ is the final O_2_ concentration (μmol/L), [O_2_]_i_ is the initial O_2_ concentration (μmol/L), V is the volume of water in each chamber (L), T is the incubation time (h), and A is the leaf area (m^2^). During the measurements, O_2_ saturation levels were periodically checked, and incubation time was adjusted to avoid O_2_ supersaturation in the chambers. P-I curves were fitted with the equation model of Smith (1936) and Talling (1957) using SigmaPlot (version 14.0, 2017 Systat Software Inc), and the maximum photosynthetic rate (Pm, μmol O_2_/m_2_/h) was calculated.

The photosynthetic pigments chlorophyll *a*, chlorophyll *b*, and total carotenoids (Car) were extracted from 200 mg of frozen leaf tissue powdered in liquid nitrogen and sodium ascorbate under dim light in 5 mL of pure acetone neutralized with calcium carbonate (Costa *et al.* 2021). The extracts were sequentially filtered with a 5.0 μm LS membrane and 0.2 μm PTFE hydrophobic filter. The photosynthetic pigments were quantified spectrophotometrically (Beckman Coulter DU-650 spectrophotometer) by reading the extracts' absorbances at 470 nm, 644.8 nm, and 661.6 nm. The concentration of photosynthetic pigments was calculated according to Lichtenthaler and Buschmann (2001).

**2.3 Antioxidant capacity, oxidative stress, and energy metabolism**

The leaf antioxidant capacity was assessed by quantifying oxygen radical absorbance capacity (ORAC) as in Costa *et al.*, (2021) after Re *et al.*, (1999), and Gillespie *et al.*, (2007). Briefly, 100 mg of frozen leaf tissue was powdered in liquid nitrogen, suspended in 3 mL of hydrochloric acid (HCl) 0.1N, kept overnight under constant shaking at 4°C, and centrifuged (4700xg, 4°C, 30 min). The supernatant was used for ORAC assays.

For the ORAC assay, 150 μL of 8.2 x 10^-5^ mM fluorescein in 75 mM potassium phosphate buffer, pH 7.4, were added to 25 μL of extract, heated to 37°C and read in a Synergy TM 4 multi-detection microplate reader (485 nm excitation filter, 20 nm bandpass, and 528 nm excitation filter, 20 nm bandpass). The reaction was initiated by adding 25 μL of freshly prepared 153 mM ABAP (lipophilic peroxyl radical generator). Results were expressed as Trolox® equivalents.

Energy metabolism was assessed by the quantification of the adenylate compounds ATP (adenosine triphosphate), ADP (adenosine diphosphate), and AMP (adenosine monophosphate), soluble sugars and starch. ATP, ADP, and AMP were extracted from frozen leaves and roots according to Liu *et al.* (2006) and quantified by isocratic HPLC analysis as in Coolen *et al.* (2008) in an Alliance Waters 2695 separation module (Milford MA, USA) with a Waters 2996 photodiode array detector and 150x4.6 mm, Kinetex 5μ C18 100Å column (Phenomenex ®). The adenylate energy charge (AEC) was calculated as in Padinha *et al.*, (2000):

AEC = (ATP + 0.5 x ADP)/(ATP + ADP+ AMP)

Soluble sugars and starch were extracted from *P. oceanica* leaves and roots. 10 mg of freeze-dried leaf tissue was powdered in a ball mill (RETSCH, MM300), extracted in ethanol 80% (10 min, 80°C), and centrifuged (2000xg, 4°C, 5 min). The supernatant was collected for soluble sugars, and the pellet for starch quantification. Before starch quantification, the pellet was washed three times with distilled water (1 mL) and incubated at 100°C for 10 min. Then, starch was hydrolyzed by a 24h incubation at 37°C in an enzyme mix (4U/mL ﻿α-amylase and 2.8U/mL amyloglucosidase in sodium acetate 0.2M, pH4). After hydrolysis, the mixture was centrifuged (750xg, 4°C, 5 min) and used for starch quantification. Soluble sugars and starch were quantified by mixing 1 mL of extract with 1 mL of 5% phenol and 5 mL of 95% sulphuric acid and reading absorbances at 450 nm and 750 nm (Beckman Coulter DU-650 spectrophotometer) and reported as Total non-structural carbohydrates (TNC) in the leaf and root. Calibration was performed using several dilutions of glucose.

**2.4 RNA extraction and sequencing**

Samples were stored overnight at 4°C to let the RNAlater solution penetrate the tissue and then stored at −20°C until RNA extraction. Total RNA was extracted from three replicates using the Aurum™ Total RNA Mini Kit (BIO-RAD). RNA purity and concentration were checked using the NanoDrop® ND-1000 Spectrophotometer (Thermo Fisher Scientific) and 1% agarose gel electrophoresis, while RNA integrity was assessed by means of 2100 BioAnalyzer (Agilent). Next-generation sequencing experiments were performed by Genomix4life S.R.L. (Baronissi, Salerno, Italy). Indexed libraries were prepared from 800 ng/ea purified RNA with Illumina Stranded mRNA Prep according to the manufacturer’s instructions. Libraries were quantified using the TapeStation 4200 (Agilent Technologies) and Qubit fluorometer (Invitrogen Co.), then pooled such that each index-tagged sample was present in equimolar amounts, with a final concentration of the pooled samples of 5nM. The pooled samples were subject to cluster generation and sequencing using an Illumina Novaseq6000 System (Illumina) in a 2x151 paired-end format. The raw sequence files generated (.fastq files) underwent quality control analysis using FastQC (http://www.bioinformatics.babraham.ac.uk/projects/fastqc).

**2.5 Differentially expressed genes (DEGs) and functional annotation analysis**

A cleaning step of the raw reads was performed using Trimmomatic (Bolger *et al.*, 2014). Low-quality reads were discarded during the cleaning step, setting the minimum quality per read at a 20 phread score. All cleaned reads were then mapped on the reference genome of *P. oceanica* (Ma *et al.*, 2024) using STAR aligner v.2.6.0c (default settings, (Dobin *et al.*, 2013). Reads count per gene for each replicate were performed using the featureCounts software (Liao *et al.*, 2014). DEGs analysis was carried out using DESeq2 (Love *et al.*, 2014) and edgeR (Robinson *et al.*, 2010), two R packages implementing two different statistical approaches. For each gene, the mean of the log2 fold change values (Log2FC) obtained with DeSeq2 and EdgeR was retrieved, setting as parameters an FDR ≤ 0.05, a P-adjusted ≤ 0.05, and and Log2 fold change ≥ |1.5|. Differential gene expression profiles were calculated for the comparisons between all treatments (heatwave, CO_2,_ and heatwave+CO_2_) and the control condition. Functional annotation of the reference transcriptome was carried out by scanning the Swiss-Prot database (Bateman, 2019) using the BLASTx software (Camacho *et al.*, 2009), setting an E-value threshold of 1e^-3^ and getting only the best hit detected. For each best hit, the SwissProt function and the related Gene Ontologies (G.O.) were retrieved. A GO enrichment analysis for all the identified DEG datasets was carried out by Ontologizer software (Bauer *et al.*, 2008), setting a p-value to p ≤ 0.05 to identify significantly enriched functional terms.

**2.6 Weighted gene co-expression network analysis (WGCNA)**

Reads counts were filtered to remove genes with less than 10 counts in all the replicates, to remove low abundancies or noise from the analysis. Filtered counts for all conditions were used to generate co-expression networks using the WGCNA package in R (Langfelder and Horvath, 2008). The adjacency matrix was constructed using a soft threshold power of 14 (**Fig. S2**). To generate networks of co-expressed genes, the blockwiseModules function (Zhang and Horvath, 2005) was used to transform the adjacency values into topological overlap measure (TOM). The different modules were identified using the dynamic tree cut algorithm (Langfelder *et al.*, 2008) setting a minimum cluster size (minModuleSize parameter) of 30 and a merging threshold function (mergeCutHeight parameter) of 0.25. To relate different studied traits with the gene networks, the module eigengenes (ME, the first principal component of each module) were correlated to 18 physiological variables. The corPvalueStudent function was selected to use a Student asymptotic p-value for correlation. Hubs genes were identified within each module based on their module membership (MM) value, also known as eigengene-based connectivity kME (intramodular connectivity; T. F. Fuller et al., 2007). This value represents the correlation between the expression level of a particular gene and the module eigengene (Horvath and Dong, 2008). Genes with higher MM values are highly connected within a particular module (Fuller *et al.*, 2011). A GO enrichment analysis for all the detected modules was carried out by Ontologizer software (Bauer *et al.*, 2008), setting a p-value to p ≤ 0.05 to identify significantly enriched co-expressed functional terms. The RPKM (reads per kilobase of transcript per million reads mapped) was considered for discussing the abundance level of key genes observed in the significant modules.

**2.7 Statistics**

Plant trait responses (Fv/Fm, Net-Photosynthesis, Dark Respiration, Total photosynthetic pigments, gross-photosynthesis, gross-photosynthesis: dark-respiration ratio (P:R Ratio), ORAC, AEC, TNC_Leaf, TNC_Roots) were analyzed with two-way ANOVA to detect significant differences between treatments and control. The model included CO_2_ (with two levels: control and high) and temperature (HW, with two levels: control and high) as fixed factors. Since AEC and TNC were measured in leaves and roots, the three-way ANOVA was applied to investigate significant differences between treatments and tissues, including CO2 (with two levels: control and high), temperature (HW, with two levels: control and high), and tissues (with two levels: leaf and roots) as fixed factors. Normality and homoscedasticity were checked using the Shapiro–Wilk and Levene's tests, and data was subsequently transformed where necessary. Tukey's HSD (honestly significant difference) test *post hoc* comparison was used whenever significant differences (P < 0.05) among treatments were detected. Statistical analyses were performed in R version 4.2.0 (R Core Team 2021).

**3. Results**

**3.1 Photo-physiological traits, respiration and pigment content**

The maximum photochemical efficiency of PSII (Fv/Fm) was negatively affected by the onset of a simulated marine heatwave (factor HW, p-value < 0.01, **Tab. S1.1**, **Fig. S3A**), while no changes were observed in gross photosynthesis (**Fig. S3b**). In detail, Fv/Fm decreased in heated seedlings when compared with control (-27.9% in HW and -28.7% in CO_2_+HW) irrespective of the CO_2_ level. The rate of photosynthesis (Net-Photosynthesis, **Fig. 2A**) doubled under high-CO_2_ (p-value < 0.001, HSD *post-hoc* test), except when combined with heatwave conditions (CO_2_+HW). Dark respiration (DR) increased significantly with heatwave conditions, except when in combination with high-CO_2_ (p-value < 0.001, HSD *post-hoc* test; **Tab. S1.1**, **Fig. 2B**). However, the grossphotosynthesis:dark-respiration ratio (P:R Ratio) increased only in high-CO_2_, while remained similar to controls in HW and CO_2_+HW (**Fig. S3C**). The leaf photosynthetic pigments content was not significantly affected by the experimental treatments (**Fig. S3D**).

**3.2 Antioxidant response and Energy metabolism**

The oxygen radical absorbance capacity (ORAC) of *P. oceanica* leaves was significantly higher in the CO_2_+HW treatment (**Fig. 3, Tab. S1.1**), while no changes were detected for HW and CO_2_. The adenylate energy charge (AEC) ratio did not show any significant trends in response to treatments in both leaves and roots (**Fig. S4A, Tab. S1.1**). However, a slight increase was observed under CO_2_+HW treatment compared to C in leaves with significant differences observed between leaves and roots (p-value < 0.01, three-way ANOVA). Total nonstructural carbohydrates (TNC) measured in leaves and roots across treatments were not affected by treatments (**Fig. S4B**).

**3.3 Differentially expressed genes (DEGs) and GO enrichment**

The largest transcriptomic response in a number of DEGs was observed in CO_2_ + HW, sharing more DEGs with HW (**Fig. 4**). Seedlings long exposed to high-CO_2_ levels (CO_2_) repressed the largest majority of genes rather than over-expressing them. This result contrasts with CO_2_+HW and HW, where the number of up-regulated and down-regulated DEGs was comparable.

*HW*: Heat shock proteins and chaperons were among the most expressed transcripts in HW treatment (18.2 kDa class I heat shock protein, Chaperone protein ClpB1), which was enriched in specific biological processes related to heat responses (*pyrimidine-containing compound metabolic process*, *response to heat* and *protein folding*; **Fig. 5A, Tab. S2, and Tab. S3**).

*CO_2_*: CO_2_ treatment was enriched in processes involved in flavonoids (*flavonoid biosynthetic* and *metabolic processes*; **Fig. 5B, Tab. S2, and Tab. S3**) and *photoprotection,* where transcripts involved in the flavonoid biosynthesis, the regulation of the cyclic electron flow (CEF) around Photosystem I, and photo-protection were downregulated (Chalcone synthase, CHS; Protein proton gradient regulation 5, PGR5; Photosystem II 22 kDa protein 1, PSBS1). Here, Vacuolar-sorting receptor and transcripts with oxidoreductase activity were the most expressed (Vacuolar-sorting receptor 6, Cytochrome P450). Transporters such as Tonoplast dicarboxylate transporter (TDT) and ABC transporter G family member 2 (ABCG2) were also down-regulated.

*CO_2_+HW*: *Chromosome organization, DNA metabolic process, and DNA methylation* were exclusively enriched in the CO_2_+HW treatment (**Fig. 5C, Tab. S2, and Tab. S3**). A key gene involved in the respiratory chain (AOX1) was the most upregulated in CO_2_+HW, including also genes regulating development and cell wall organization (MIZ1, Protein MIZU-KUSSEI 1, and PME11, Putative pectinesterase 11).

**3.4 Gene co-expression network analysis (WGCNA)**

The weighted gene co-expression network analysis (WGCNA) was performed to investigate genes associated with physiological traits. The WGCNA identified ten co-expressed gene modules (labeled in different colors), shown in **Fig. 6.** Among these, six modules (red, turquoise, yellow, blue, black, and purple) were significantly correlated to physiological traits (R ≥ 0.6; P ≤ 0.05, **Fig. 7**). Red and turquoise modules were positively correlated with ORAC (MEred, R = 0.61, P = 0.03; MEturquoise, R = 0.72, P = 0.008). The blue module was negatively correlated with Fv/Fm (R = -0.6, P = 0.04). Additionally, negative correlations were observed between the black module and dark respiration (Rd; R = -0.61, P = 0.03), as well as between purple and carbon balance (P_R; R = -0.65, P = 0.02). The yellow module was positively correlated with dark respiration (Rd; R = 0.6, P = 0.04;).

**3.5 Central genes significantly correlated with physiological traits modules**

The two major significant modules, including the largest number of co-expressed transcripts, were the blue (n = 4216 genes) and the turquoise (n = 4903 genes) modules. Central genes (*i.e.* hub genes) that have a high degree of connectivity (*i.e.* MM > 0.95) and are related to enriched terms of interest are included in **Tab. S4** and **Tab. S5**.

*Modules correlated with oxygen radical absorbance capacity (ORAC)*

The turquoise module included a total of 1081 central genes (MM > 0.95, **Tab. S4)**. Among these, important genes with antioxidant activity were modulated in the CO_2_+HW treatment (COX11, OCP3, and FDX3; **Fig. 7**, **Tab. S6**). Central genes were also identified by selecting significant GO-enriched terms within the module. In this case, *energy derivation by oxidation of organic compounds* and *carboxylic acid catabolic process* were enriched in the module. Associated genes are important mitochondrial electron transport chain (mETC) regulators, like cytochromes, NADH dehydrogenases, and alternative oxidases (**Tab. S4** and **Tab. S5**).

The red module included very few central genes (MM > 0.95) in comparison to the turquoise one. Several hub genes were implicated in the oxidative and general stress response (6-phosphogluconate dehydrogenase decarboxylating 2 PGD2, E3 ubiquitin-protein ligase RGLG2, **Tab. S4**) which were particularly modulated in the CO_2_+HW treatment (**Fig. 7, Tab. S2**). Other genes involved in the *detoxification* and *regulation of response to oxidative stress* were identified (*i.e.* U17, Glutathione S-transferase F8, and ABC1K7, Protein activity of bc1 complex kinase 8, chloroplastic, **Tab. S4** and **Tab. S5**).

*Modules correlated with Respiration (Rd) and Photosynthesis - Respiration Ratio (P: R)*

In the black module, which was negatively correlated with Rd and P: R, significant genes related to photo-respiratory activities were modulated in temperature treatments (HW and CO_2_+HW), including Glyceraldehyde-3-phosphate dehydrogenase, Glutaredoxin-C4, and Probable sarcosine oxidase (**Tab. S4**). *Response to heat, photomorphogenesis*, and *protein folding* were among the most enriched biological processes. Interestingly, several terms related to the flowering process were also enriched (*photoperiodism – flowering*, *inflorescence development*, *inflorescence meristem growth*). Transcripts included in these terms were particularly modulated under HW treatment (e.g. Transcription factor GHD7, Casein kinase 1-like protein HD16, Protein heading date 3B). Several transcripts related to mitochondria were also included in this module (Mitochondrial phosphate carrier protein 1; Heat shock protein 90-6, mitochondrial; 10 kDa chaperonin, mitochondrial; Heat shock 70 kDa protein, mitochondrial, **Tab. S4** and **Tab. S5**).

The purple module was the least representative one in terms of central genes. Different transcription factors associated with stress response, such as Zinc finger A20 and AN1 domain-containing stress-associated protein and Heat stress transcription factor B-1, were modulated in HW treatment (**Fig. 7, Tab. S4**). Accordingly, among the most significant enriched terms were *responses to stress* and *macromolecule modification*.

Contrary to black and purple modules, yellow module was positively correlated with only Rd (**Fig. 6**, **7**). Among the central genes found in the yellow module, are EX2 (Protein EXECUTER 2, chloroplastic) which is involved in the signaling pathway mediated by singlet oxygen, and MBS1 (Protein methylene blue sensitivity 1) which is required for the acclimation process to reactive oxygen species (ROS). Here, genes involved in plant development were also detected (CYT1, Mannose-1-phosphate guanylyltransferase 1; RE, Protein RETICULATA, chloroplastic) (**Tab. S6**). Structural transcripts forming protein complexes such as mitochondrial membrane ATP synthase (ATP synthase 6 kDa subunit, mitochondrial; ATP synthase subunit O, mitochondrial), NADH dehydrogenases, and intermembrane chaperones were also found. In addition, several biological processes related to transport activity, apoptotic processes, and protein degradation were enriched (*nitrogen compound transport,* *peptide metabolic process*, *regulation of anoikis*, and *response to oxidative stress*) (**Tab. S4** and **Tab. S5**).

*Module correlated with Photochemical efficiency (Fv/Fm)*

The blue module was the only one that negatively correlated with Fv/Fm (**Fig. 6**, **7**). Here, transcripts with antioxidant activity like Superoxide dismutase [Cu-Zn] 2 (SODCC.2), Oxidation resistance protein 1 (Oxr1), and Peroxisomal 2, 4-dienoyl-CoA reductase were observed. Among enriched terms, different regulatory processes appeared to be modulated, such as *DNA repair, RNA modification, methylation*, and *histone modification*. Some transcripts included in these terms were repressors of the transcription (i.e. Ethylene-responsive transcription factor ABI4, DELLA protein GAI) (**Tab. S2**).

**3.6.** **Intersecting DEGs and WGCNA results (key genes in stress responses)**

Differentially expressed genes found for each treatment (HW, CO_2_, CO_2_ + HW) overlapping with all transcripts included in significant modules (WGCNA, **Fig. S5, and Tab. S7)** were explored to identify key regulators of single and multiple stressors exposure. The results showed that 484 DEGs were related to specific physiological traits (*i.e.* ORAC, Fv/Fm, Rd, P: R). Among these, 216 DEGs were uniquely regulated in the CO_2_+HW treatment, followed by HW (n = 186) and CO_2_ (n = 82). The former included several genes involved in DNA and chromatin modifications such as ATP-dependent DNA helicase DDM1, DNA (cytosine-5)-methyltransferase 1A, and Lysine-specific demethylase JMJ25, which were overexpressed in CO_2_+HW. Other important genes involved in DNA repair mechanisms and oxidative stress response were expressed under CO_2_+HW treatment (DNA repair protein RAD50, Inactive poly [ADP-ribose] polymerase RCD1). Moreover, the majority of resulting genes were shared between HW and CO_2_ + HW. Contrarily, genes with antioxidant activity were repressed in the HW treatment (Polyphenol oxidase, chloroplastic (PPO), Peroxidase 12). Here, several heat shock proteins were also expressed (Heat shock 70 kDa protein 14, 15, 81-2, Heat stress transcription factor A-2b). Moreover, a key gene involved in the regulation of the circadian clock and auxin pathways (reveille 1) was largely down-regulated in HW, followed by Polyphenol oxidase, chloroplastic (PPO), and Glutelin type-D 1 (GLUD1). In the CO_2_ treatment, genes involved in the electron transport (PSBS1, Photosystem II 22 kDa protein 1, chloroplastic), cell wall organization (XTH23, Probable xyloglucan endotransglucosylase/hydrolase protein 23), and transferase activity (HPCA1, Leucine-rich repeat receptor protein kinase) were down-regulated.

**4. Discussion**

In a future scenario of OA, *Posidonia oceanica* will be more capable of coping with the observed and forecasted increase of MHWs frequency. Our results, in fact, show that increased levels of CO_2_ in seawater will be a positive factor in enhancing the physiological response of *P. oceanica* seedlings to marine heat waves. Under current CO_2_ concentrations, anomalous high temperatures induced stress on *P. oceanica* seedlings and altered their photosynthetic and respiratory rates, potentially eroding their health and energy status. However, when seedlings were grown under OA conditions, they were able to regulate the transcriptomic machinery to counteract the effects of heat stress by regulating their metabolic rates and enhancing their antioxidant defense. This is because elevated CO_2_ concentrations, rather than negatively affecting *P. oceanica* seedlings, provide them with greater “resources” in the form of increased carbon accumulation and energy supply that can be used to cope with heat stress conditions, which is reasonable since seagrasses tend to be carbon-limited under natural conditions (Pajusalu *et al.*, 2016). Under elevated CO_2_ conditions and no other nutrient limitations, the increased concentration of carbon allows seedlings to enhance their photosynthetic capacity, leading to greater carbon fixation and accumulation, which in turn contributes to withstand thermal stress. This is the first study conducted on *P. oceanica* seedlings that confirms the beneficial effect of CO_2_ availability in improving seagrass responses to thermal stress. This has been evident in this study, where anomalous warming events cause deleterious effects on *P. oceanica* seedlings (reduction of Fv/Fm, increased respiration rate and the expression of specific genes), whereas the co-occurrence of acidification of ocean conditions in the coming decades could reverse these negative effects, making the early life stages of seagrasses more resilient to warming than previously thought. OA could favor recruitment of *P. oceanica* seedlings, whose amount is predicted to increase under increased MHW frequency and duration. Their higher sensitivity to temperature increase, in respect to adult plants (Rinaldi *et al.*, 2023), pose important doubts on the evolutionary effectiveness of the increased flowering response to global warming.

Under current CO2 conditions, *P. oceanica* seedlings exposed to heat showed alterations as already described in heat-stressed seedlings and adults of the species (Dattolo et al., 2017; Marín-Guirao et al., 2016; Pazzaglia et al., 2020; Pazzaglia, et al., 2022; Stipcich, et al., 2022). These alterations include reductions in photosynthetic yields, increases in respiratory metabolic rates at the physiological level, and a strong transcriptomic regulation of HSPs and molecular chaperones at the molecular level. As a consequence of these modifications, the energetic state of plants deteriorates, which in the long term can lead to death (Marín-Guirao *et al.*, 2016, 2017; Ruocco *et al.*, 2019d). Surprisingly, seedlings that experienced anomalous warming after months of growth in CO_2_-enriched future ocean conditions were able to maintain their respiratory metabolic rate unchanged. Respiratory homeostasis under warming seawater conditions is a functional trait associated with heat tolerance in seagrasses (Collier *et al.*, 2011; Marín-Guirao *et al.*, 2018), which could be associated with transcriptional regulations at the mitochondrial level (Marín-Guirao *et al.*, 2017; Nguyen *et al.*, 2021). Seedlings heated under OA conditions indeed show a strong induction of the alternative oxidase (AOX) pathway of the mitochondrial electron transport chain, a response with potential implications for supporting homeostasis in carbon and energy metabolism in stressed plants (Vanlerberghe, 2013). Modifications in the activity of the alternative oxidase (AOX) have been described under marine heatwave conditions in *P. oceanica* (Pazzaglia et al., 2022; Ruocco et al., 2019; Traboni et al., 2018a; Tutar et al., 2017) and in terrestrial plants with implications on leaf respiratory rates (Scafaro *et al.*, 2021). In addition to the regulation of cellular respiratory metabolism, one of the main functions of the AOX pathway is the control of ROS production in mitochondria, which increases in plants under conditions of stress and accelerated metabolism (Marquez-Acevedo *et al.*, 2023). In our case, seedlings heated in CO_2_-enriched conditions showed an antioxidant capacity (ORAC) twice as high as seedlings in normal CO_2_ conditions. Gene co-expression network analysis (WGCNA) identifies transcriptomic modulation involved in this increased antioxidant competence, where ORAC-correlated modules contained a number of core genes involved in the regulation of oxidative stress and antioxidant defense (e.g. GSTF8, FDX3, romo1, OCP3). Therefore, in addition to controlling their respiration rates and ROS production, seedlings grown in future OA conditions were able to activate a higher antioxidant defense and a stronger transcriptomic regulation that was directly involved in the regulation of phenotypic adjustments to cope with anomalous temperatures than seedlings in current pH conditions. This ability could be associated with a better energetic and fitness state acquired while growing under high CO_2_ conditions. Therefore, long-term exposure to CO_2_ enriched environment has the potential to mitigate the negative effect abiotic stresses by acting as a fertilizer in several plant species (i.e. CO_2_ fertilizer effect; Kazemi et al., 2018; Shokat et al., 2021). Stress-mitigation under elevated CO_2_ was already observed in plants as the result of improved ROS detoxification through the regulation of antioxidants (Ghasemzadeh *et al.*, 2010; Zinta *et al.*, 2014). Moreover, growth in a CO_2_-rich environment can trigger a priming effect in plants through the activation of metabolic processes and redox signaling, potentially improving their resistance to infections (Mhamdi and Noctor, 2016). This may increase plasticity regulating the response machinery more efficiently in the presence of the additional stressor (Liu *et al.*, 2022). Seedlings under these conditions showed much higher photosynthetic rates than seedlings under current CO_2_ conditions, resulting in carbon balances (P:R ratio) twice as high. The mechanism employed by *P. oceanica* seedlings to take advantage of the increased CO_2_ availability appears to be the inactivation of non-photochemical quenching, one of the main photoprotection mechanisms involved in the dynamic regulation of photosynthesis and which has been shown to operate in the species (Marín-Guirao et al., 2013). This process is linked to the xanthophyll cycle and requires for its activation the acidification of the thylakoid lumen that occurs under high-light conditions together with the encoding of the PSBS protein (Li *et al.*, 2000; Niyogi *et al.*, 2005). In seedlings under OA conditions, the strong downregulation of the gene encoding the PSBS protein and the PGR5 gene, which contributes to creating a proton gradient across thylakoid membranes (Wang *et al.*, 2014), supports this hypothesis. This higher productivity, however, is not reflected in higher carbohydrate content in leaf tissues, possibly because these seedlings were still attached to their seeds. *Posidonia oceanica* seeds contribute to sustaining seedling development until about one year after germination, thanks to the large amount of resources they store and their ability to photosynthesize (Balestri *et al.*, 2009; Rinaldi *et al.*, 2023). It is possible that the sugars produced by seedlings were stored in the seeds since the rhizome, which is the reserve organ of the species, had not yet developed. However, the content of non-structural carbohydrates in seeds was not analyzed. This possibility is supported by the fact that the biomass of *P. oceanica* seedlings maintained under high CO_2_ concentration for three months was almost 2-fold higher than control seedlings but without differences in leaf and root biomass (Hernán *et al.*, 2016). Similarly, adult seagrasses can also exhibit an increase in belowground biomass, reflecting increased storage of reserve compounds (Palacios and Zimmerman, 2007). The increased productivity due to months under CO_2_-enriched conditions, with the higher amount of resources stored in the seeds seems to have provided the seedlings with resources to better cope with an anomalous warming event. Moreover, seedlings’ roots were already developed suggesting that reserves were used to build fibers favoring the seedling growth. On the other hand, despite their ability to control respiration, heated seedlings under OA conditions showed photosynthetic alterations similar to seedlings heated under current-day CO_2_ concentration. This is because the photochemical process is impacted by temperature increases, as already described in previous studies (Marín-Guirao *et al.*, 2017; Ontoria *et al.*, 2019; Nguyen *et al.*, 2020). A slight reduction in photochemical efficiency does not significantly affect net photosynthetic rates, probably due to a modification of the activity of alternative O_2_-consuming metabolic pathways (photorespiration, Mehler reaction, cellular respiration, chlororespiration and mitochondrial alternative oxidase pathway) as described for this and other seagrass species subjected to abiotic stress, including heat, salinity or high light (Touchette and Burkholder, 2000; Silva *et al.*, 2013). However, the prolonged exposure to elevated CO_2_ equipped the seedlings to better cope with stress by activating specific regulatory pathways (i.e. antioxidants, photoprotection), thereby maintaining a stable carbon balance. Noteworthy, genes involved in DNA and chromatin modifications like DDM1 and JMJ25 were over-expressed in seedling grown under increased CO2 concentration. Their expression patterns correlated with antioxidant capacity, and photochemical efficiency, respectively. Besides DNA methylation being poorly investigated in *P. oceanica*, recent evidence has described a key regulatory and dynamic role of the process in response to heat stress (Pazzaglia *et al.*, 2023). In particular, DDM1 encodes for DNA (cytosine-5)-methyltransferase 1A which is a Snf2 remodeler essential for normal DNA methylation in plants (Zemach *et al.*, 2013). Recently, the increase in DNA methylation levels was described during seed development in plants, and potentially related to the antioxidant capacity under temperature changes (Cui *et al.*, 2022). A similar relationship was also observed for Lysine-specific demethylase JMJ25, which is implicated in the regulation of anthocyanin biosynthetic genes. This group of flavonoids is involved in the response to abiotic stress by scavenging ROS and reducing oxidative stress. These results opened further questions on the potential regulatory role played by DNA methylation in mediating stress responses in seagrasses.

In conclusion, this study revealed that the future increased availability of CO_2_ in the oceans could foster productivity of *Posidonia oceanica* meadows, making young plants more resilient against global environmental stressors and local human-derived threats and better equipped for developing in adult plants. Further multiple-stressors assessments in different seagrass species are needed, considering the effects of OA on the whole seagrass ecosystem including the associated communities. Although our study did not include sampling before CO₂ treatment or between the CO₂ and heatwave phases, future studies with time-series sampling could better track metabolic changes providing a more comprehensive understanding of the acclimation process and stress responses. Additionally, integrating more ecologically realistic simulations—such as natural patterns of temperature fluctuation and variable stress exposure across time—would enhance our ability to predict long-term plant performance under future climate scenarios. Nevertheless, considering the paramount importance of ecosystem services provided by seagrass meadows and their role in enhancing human health and human livelihood along the coastline, these results represent a positive message and a potential ray of light for the future.

**Supplementary data**

The following supplementary data are available at JXB online.

**Fig. S1** Photosynthetic performance (FvFm) during the experimental phase.

**Fig. S2** Scale independence and mean connectivity analysis.

**Fig. S3** Photosynthetic performance metrics in seedlings under treatments.

**Fig. S4** Metabolic indicators in leaves and roots under treatments.

**Fig. S5** Number of genes overlapped with weighted co-expression analysis (WGCNA) and differentially expressed genes (DEGs).

**Table S1** Two-way ANOVA analysis for the photo-physiological and biochemical traits measured in seedlings exposed treatments.

**Table S2** Differentially Expressed Genes (DEGs) across treatments.

**Table S3** GO enrichment analysis performed for seedlings exposed to treatments.

**Table S4** Transcripts included in the most significant modules.

**Table S5** GO enrichment in significant modules.

**Table S6** Hub genes selected from significant modules.

**Table S7** Transcripts that were both differentially expressed (DEG) and significantly correlated with plant traits.

**Funding**

This work was supported by the project Marine Hazard, PON03PE_00203_1 (Ministry of Education, University and Research, MUR, Italy), by the project Assemble Plus (EU-H2020, Grant No. 730984). The work of LMG was partially supported by the GRASSREC 683 project, PID2020-118144RB-C31 (Spanish Ministry of Science and Innovation). JP was supported during the experimental phase by the Ph.D. fellowship funded by the University of Trieste (Italy) and the Stazione Zoologica Anton Dohrn (Italy). JP and GP were also supported by the National Recovery and Resilience Plan (NRRP), Mission 4 Component 2 Investment 1.4 - Call for tender No. 3138 of 16 December 2021, rectified by Decree n.3175 of 18 December 2021 of Italian Ministry of University and Research funded by the European Union – NextGenerationEU (project code CN_00000033, Concession Decree No. 1034 of 17 June 2022 adopted by the Italian Ministry of University and Research, CUP C63C22000520001, Project title “National Biodiversity Future Center - NBFC” ).

The study also received Portuguese National Funds from FCT-Fundação para a Ciência e a Tecnologia through projects UIDB/04326/2020 (DOI:10.54499/UIDB/04326/2020), UIDP/04326/2020 (DOI:10.54499/UIDP/04326/2020), and LA/P/0101/2020 (DOI:10.54499/LA/P/0101/2020).

**Acknowledgments**We acknowledge V.M. Giacalone and F. Badalamenti for collecting P. oceanica seeds and maintaining them at the CNR-IAS facility until the start of the experimental phase.

**Author contributions**

JP-JS-LMG-GP: conceptualization; JP-JS-IB-KP-MC-LA: Data Curation, Investigation; JP-JS-IS-LA: Formal Analysis; JP: Validation, Writing – Original Draft Preparation; JP-JS-IB-KP-LA-LMG-GP: Writing – Review & Editing.

**Competing interests**

The authors declare no conflict of interest

**Data availability**

Raw reads are freely available at the Sequence Read Archive (SRA) partition of NCBI database (PRJNA577416, reviewer link: <https://dataview.ncbi.nlm.nih.gov/object/PRJNA1131296?reviewer=jc33uev3ct5p6k79i48e8nrm53>) **References**

**Amrine KCH, Blanco-Ulate B, Cantu D**. 2015. Discovery of core biotic stress responsive genes in Arabidopsis by weighted gene co-expression network analysis. PLoS ONE **10**, 1–20.

**Anderson JT, Mitchell-Olds T**. 2011. Ecological genetics and genomics of plant defences: Evidence and approaches. Functional Ecology **25**, 312–324.

**Apostolaki ET, Vizzini S, Hendriks IE, Olsen YS**. 2014. Seagrass ecosystem response to long-term high CO2 in a Mediterranean volcanic vent. Marine Environmental Research **99**, 9–15.

**Balestri E, Gobert S, Lepoint G, Lardicci C**. 2009. Seed nutrient content and nutritional status of Posidonia oceanica seedlings in the northwestern Mediterranean Sea. Marine Ecology Progress Series **388**, 99–109.

**Bateman A**. 2019. UniProt: a worldwide hub of protein knowledge. Nucleic Acids Research **47**, D506–D515.

**Bauer S, Grossmann S, Vingron M, Robinson PN**. 2008. Ontologizer 2.0—a multifunctional tool for GO term enrichment analysis and data exploration. Bioinformatics **24**, 1650–1651.

**Beca-Carretero P, Guihéneuf F, Marín-Guirao L, Bernardeau-Esteller J, García-Muñoz R, Stengel DB, Ruiz JM**. 2018. Effects of an experimental heat wave on fatty acid composition in two Mediterranean seagrass species. Marine Pollution Bulletin **134**, 27–37.

**Bolger AM, Lohse M, Usadel B**. 2014. Trimmomatic: a flexible trimmer for Illumina sequence data. Bioinformatics **30**, 2114–2120.

**Camacho C, Coulouris G, Avagyan V, Ma N, Papadopoulos J, Bealer K, Madden TL**. 2009. BLAST+: architecture and applications. BMC Bioinformatics 2009 10:1 **10**, 1–9.

**Collier CJ, Uthicke S, Waycott M**. 2011. Thermal tolerance of two seagrass species at contrasting light levels: Implications for future distribution in the Great Barrier Reef. Limnology and Oceanography doi: 10.4319/lo.2011.56.6.2200.

**Costa MM, Silva J, Barrote I, Santos R**. 2021. Heatwave Effects on the Photosynthesis and Antioxidant Activity of the Seagrass Cymodocea nodosa under Contrasting Light Regimes. Oceans 2021, Vol. 2, Pages 448-460 **2**, 448–460.

**Cox TE, Schenone S, Delille J, Díaz-Castañeda V, Alliouane S, Gattuso J-P, Gazeau F**. 2015. Effects of ocean acidification on *Posidonia oceanica* epiphytic community and shoot productivity. (B Silliman, Ed.). Journal of Ecology **103**, 1594–1609.

**Cui C, Wang Z, Su Y, Wang T**. 2022. Antioxidant Regulation and DNA Methylation Dynamics During Mikania micrantha Seed Germination Under Cold Stress. Frontiers in Plant Science **13**, 856527.

**Dattolo E, Marín‐Guirao L, Ruiz JM, Procaccini G**. 2017. Long‐term acclimation to reciprocal light conditions suggests depth‐related selection in the marine foundation species Posidonia oceanica. Ecology and Evolution **7**, 1148–1164.

**Dattolo E, Ruocco M, Brunet C, Lorenti M, Lauritano C, D’Esposito D, de Luca P, Sanges R, Mazzuca S, Procaccini G**. 2014. Response of the seagrass Posidonia oceanica to different light environments: Insights from a combined molecular and photo-physiological study. Marine Environmental Research **101**, 225–236.

**Deguette A, Barrote I, Silva J**. 2022. Physiological and morphological effects of a marine heatwave on the seagrass Cymodocea nodosa. Scientific Reports **12**, 1–13.

**Dobin A, Davis CA, Schlesinger F, Drenkow J, Zaleski C, Jha S, Batut P, Chaisson M, Gingeras TR**. 2013. STAR: Ultrafast universal RNA-seq aligner. Bioinformatics **29**, 15–21.

**Fuller TF, Ghazalpour A, Aten JE, Drake TA, Lusis AJ, Horvath S**. 2007. Weighted gene coexpression network analysis strategies applied to mouse weight. Mammalian genome : official journal of the International Mammalian Genome Society **18**, 463–472.

**Fuller T, Langfelder P, Presson A, Horvath S**. 2011. Review of Weighted Gene Coexpression Network Analysis. Handbook of Statistical Bioinformatics, 369–388.

**Ghasemzadeh A, Jaafar HZE, Rahmat A**. 2010. Elevated Carbon Dioxide Increases Contents of Flavonoids and Phenolic Compounds, and Antioxidant Activities in Malaysian Young Ginger (Zingiber officinale Roscoe.) Varieties. Molecules **15**, 7907.

**Gillespie KM, Chae JM, Ainsworth EA**. 2007. Rapid measurement of total antioxidant capacity in plants. Nature protocols **2**, 867–870.

**Gu R, Statton J, Rahmawati S, Hovey R, Zhou Y, Tang J, Yu S, Kendrick GA**. 2022. Seed bank dynamics and quality in the seagrass Halophila ovalis along estuarine salinity gradients—a case in the Swan-Canning Estuary. Frontiers in Marine Science **9**, 1–13.

**Guerrero-Meseguer L, Cox TE, Sanz-Lázaro C, Schmid S, Enzor LA, Major K, Gazeau F, Cebrian J**. 2020*a*. Does Ocean Acidification Benefit Seagrasses in a Mesohaline Environment? A Mesocosm Experiment in the Northern Gulf of Mexico. Estuaries and Coasts **43**, 1377–1393.

**Guerrero-Meseguer L, Marín A, Sanz-Lázaro C**. 2017. Future heat waves due to climate change threaten the survival of P. oceanica seedlings. Environmental Pollution **230**, 40–45.

**Guerrero-Meseguer L, Marín A, Sanz-Lázaro C**. 2020*b*. Heat wave intensity can vary the cumulative effects of multiple environmental stressors on Posidonia oceanica seedlings: Heat waves override other stressors. Marine Environmental Research **159**.

**Guerrero-Meseguer L, Sanz-Lázaro C, Marín A**. 2018. Understanding the sexual recruitment of one of the oldest and largest organisms on Earth, the seagrass Posidonia oceanica. PLoS ONE **13**, 1–20.

**Halpern BS, Frazier M, Potapenko J, *et al.*** 2015. Spatial and temporal changes in cumulative human impacts on the world’s ocean. Nature Communications **6**, 1–7.

**Hansen PB, Ruud AK, de los Campos G, Malinowska M, Nagy I, Svane SF, Thorup-Kristensen K, Jensen JD, Krusell L, Asp T**. 2022. Integration of DNA Methylation and Transcriptome Data Improves Complex Trait Prediction in Hordeum vulgare. Plants **11**.

**Hendriks IE, Olsen YS, Ramajo L, Basso L, Steckbauer A, Moore TS, Howard J, Duarte CM**. 2014. Photosynthetic activity buffers ocean acidification in seagrass meadows. Biogeosciences **11**, 333–346.

**Hernán G, Ortega MJ, Gándara AM, Castejón I, Terrados J, Tomas F**. 2017. Future warmer seas: increased stress and susceptibility to grazing in seedlings of a marine habitat-forming species. Global Change Biology **23**, 4530–4543.

**Hernán G, Ramajo L, Basso L, Delgado A, Terrados J, Duarte CM, Tomas F**. 2016. Seagrass (Posidonia oceanica) seedlings in a high-CO2 world: From physiology to herbivory. Scientific Reports **6**, 1–12.

**Horvath S, Dong J**. 2008. Geometric Interpretation of Gene Coexpression Network Analysis. PLOS Computational Biology **4**, e1000117.

**Jahnke M, D’Esposito D, Orrù L, Lamontanara A, Dattolo E, Badalamenti F, Mazzuca S, Procaccini G, Orsini L**. 2019. Adaptive responses along a depth and a latitudinal gradient in the endemic seagrass Posidonia oceanica. Heredity **122**, 233–243.

**Ju F, Sun L, Xiong C, Wang Z, Yu H, Pang J, Bai H, Zhao W, Zhou Z, Chen B**. 2023. Weighted gene co-expression network analysis revealed the key pathways and hub genes of potassium regulating cotton root adaptation to salt stress. Frontiers in Plant Science **14**, 1–15.

**Kaldy JE, Brown CA, Pacella SR**. 2022. Carbon limitation in response to nutrient loading in an eelgrass mesocosm: influence of water residence time. Marine Ecology Progress Series **689**, 1–17.

**Kazemi S, Eshghizadeh HR, Zahedi M**. 2018. Responses of Four Rice Varieties to Elevated CO2 and Different Salinity Levels. Rice Science **25**, 142–151.

**Koch M, Bowes G, Ross C, Zhang XH**. 2013. Climate change and ocean acidification effects on seagrasses and marine macroalgae. Global Change Biology **19**, 103–132.

**Langfelder P, Horvath S**. 2008. WGCNA: An R package for weighted correlation network analysis. BMC Bioinformatics **9**.

**Langfelder P, Zhang B, Horvath S**. 2008. Defining clusters from a hierarchical cluster tree: the Dynamic Tree Cut package for R. **24**, 719–720.

**Lee J, Hughes BB, Kroeker KJ, Owens A, Wong C, Micheli F**. 2022. Who wins or loses matters: Strongly interacting consumers drive seagrass resistance under ocean acidification. Science of The Total Environment **808**, 151594.

**Li XP, Björkman O, Shih C, Grossman AR, Rosenquist M, Jansson S, Niyogi KK**. 2000. A pigment-binding protein essential for regulation of photosynthetic light harvesting. Nature **403**, 391–395.

**Liao Y, Smyth GK, Shi W**. 2014. featureCounts: an efficient general purpose program for assigning sequence reads to genomic features. Bioinformatics **30**, 923–930.

**Listiawati V, Kurihara H**. 2021. Ocean warming and acidification modify top-down and bottom-up control in a tropical seagrass ecosystem. Scientific Reports 2021 11:1 **11**, 1–10.

**Liu H, Able AJ, Able JA**. 2022. Priming crops for the future: rewiring stress memory. Trends in Plant Science **27**, 699–716.

**Love MI, Huber W, Anders S**. 2014. Moderated estimation of fold change and dispersion for RNA-seq data with DESeq2. Genome Biology 2014 15:12 **15**, 1–21.

**Lowell A, Infantes E, West L, Puishys L, Hill CEL, Ramesh K, Peterson B, Cebrian J, Dupont S, Cox TE**. 2021. How Does Ocean Acidification Affect the Early Life History of Zostera marina? A Series of Experiments Find Parental Carryover Can Benefit Viability or Germination. Frontiers in Marine Science **8**, 1–16.

**Ma X, Vanneste S, Chang J, *et al.*** 2024. Seagrass genomes reveal ancient polyploidy and adaptations to the marine environment. Nature Plants doi: 10.1038/s41477-023-01608-5.

**Marín-Guirao L, Bernardeau-Esteller J, García-Muñoz R, Ramos A, Ontoria Y, Romero J, Pérez M, Ruiz JM, Procaccini G**. 2018. Carbon economy of Mediterranean seagrasses in response to thermal stress. Marine Pollution Bulletin **135**, 617–629.

**Marín-Guirao L, Dattolo E, Ruiz JM, Procaccini G**. 2015. Differential tolerance and resilience of Mediterranean seagrasses to short-term heat stress. 7287.

**Marín-Guirao L, Entrambasaguas L, Dattolo E, Ruiz JM, Procaccini G**. 2017. Molecular Mechanisms behind the Physiological Resistance to Intense Transient Warming in an Iconic Marine Plant. Frontiers in Plant Science, 8–1142.

**Marín-Guirao L, Entrambasaguas L, Ruiz JM, Procaccini G**. 2019. Heat-stress induced flowering can be a potential adaptive response to ocean warming for the iconic seagrass Posidonia oceanica. Molecular Ecology **28**, 2486–2501.

**Marín-Guirao L, Ruiz JM, Dattolo E, Garcia-Munoz R, Procaccini G**. 2016. Physiological and molecular evidence of differential short-Term heat tolerance in Mediterranean seagrasses. Scientific Reports **6**, 1–13.

**Marín-Guirao L, Sandoval-Gil JM, Bernardeau-Esteller J, Ruíz JM, Sánchez-Lizaso JL**. 2013. Responses of the Mediterranean seagrass Posidonia oceanica to hypersaline stress duration and recovery. Marine Environmental Research **84**, 60–75.

**Marín‐Guirao L, Entrambasaguas L, Ruiz JM, Procaccini G**. 2019. Heat‐stress induced flowering can be a potential adaptive response to ocean warming for the iconic seagrass Posidonia oceanica. Molecular Ecology, 1–16.

**Marquez-Acevedo AS, Hood WR, Collier RJ, Skibiel AL**. 2023. Graduate Student Literature Review: Mitochondrial response to heat stress and its implications on dairy cattle bioenergetics, metabolism, and production. Journal of Dairy Science **106**, 7295–7309.

**Mhamdi A, Noctor G**. 2016. High CO2 Primes Plant Biotic Stress Defences through Redox-Linked Pathways. Plant Physiology **172**, 929.

**Monnier B, Pergent G, Mateo MÁ, Clabaut P, Pergent-Martini C**. 2022. Quantification of blue carbon stocks associated with Posidonia oceanica seagrass meadows in Corsica (NW Mediterranean). Science of The Total Environment **838**, 155864.

**Nguyen HM, Kim M, Ralph PJ, Marín-Guirao L, Pernice M, Procaccini G**. 2020. Stress memory in seagrasses: first insight into the effects of thermal priming and the role of epigenetic modifications. Frontiers in Plant Science **11**, 494.

**Nguyen HM, Ralph PJ, Marín‐Guirao L, Pernice M, Procaccini G**. 2021. Seagrasses in an era of ocean warming: a review. Biological Reviews **96**, 2009–2030.

**Niyogi KK, Li XP, Rosenberg V, Jung HS**. 2005. Is PsbS the site of non-photochemical quenching in photosynthesis? Journal of Experimental Botany **56**, 375–382.

**Olivé I, Silva J, Lauritano C, Costa MM, Ruocco M, Procaccini G, Santos R**. 2017. Linking gene expression to productivity to unravel long-and short-term responses of seagrasses exposed to CO2 in volcanic vents. Scientific Reports **7**, 1–12.

**Oliver ECJ, Donat MG, Burrows MT, *et al.*** 2018. Longer and more frequent marine heatwaves over the past century. Nature Communications **9**, 1–12.

**Ondiviela B, Losada IJ, Lara JL, Maza M, Galván C, Bouma TJ, van Belzen J**. 2014. The role of seagrasses in coastal protection in a changing climate. Coastal Engineering **87**, 158–168.

**Ontoria Y, Cuesta-Gracia A, Ruiz JM, Romero J, Pé Rez M**. 2019. The negative effects of short-term extreme thermal events on the seagrass Posidonia oceanica are exacerbated by ammonium additions. doi: 10.1371/journal.pone.0222798.

**Pacella SR, Brown CA, Waldbusser GG, Labiosa RG, Hales B**. 2018. Seagrass habitat metabolism increases short-term extremes and long-term offset of CO2 under future ocean acidification. Proceedings of the National Academy of Sciences of the United States of America **115**, 3870–3875.

**Padinha C, Santos R, Brown MT**. 2000. Evaluating environmental contamination in Ria Formosa (Portugal) using stress indexes of Spartina maritima. Marine Environmental Research **49**, 67–78.

**Pajusalu L, Martin G, Põllumäe A, Paalme T**. 2016. The influence of CO2 enrichment on net photosynthesis of seagrass zostera marina in a brackish water environment. Frontiers in Marine Science **3**, 239.

**Palacios SL, Zimmerman RC**. 2007. Response of Eelgrass Zostera marina to CO2 Enrichment: Possible Impacts of Climate Change and Potential for Remediation of Coastal Habitats. Marine Ecology Progress Series **109**, 1–13.

**Pazzaglia J, Badalamenti F, Bernardeau-Esteller J, Ruiz JM, Giacalone VM, Procaccini G, Marín-Guirao L**. 2022*a*. Thermo-priming increases heat-stress tolerance in seedlings of the Mediterranean seagrass P. oceanica. Marine Pollution Bulletin **174**, 113164.

**Pazzaglia J, Dattolo E, Ruocco M, Santillán-Sarmiento A, Marin-Guirao L, Procaccini G**. 2023. DNA methylation dynamics in a coastal foundation seagrass species under abiotic stressors. Proceedings of the Royal Society B: Biological Sciences **290**, 20222197.

**Pazzaglia J, Reusch TBH, Terlizzi A, Marín-Guirao L, Procaccini G**. 2021. Phenotypic plasticity under rapid global changes: The intrinsic force for future seagrasses survival. Evolutionary Applications **00**, 1–21.

**Pazzaglia J, Santillán-sarmiento A, Helber SB, Ruocco M, Terlizzi A, Marín-guirao L, Procaccini G**. 2020. Does warming likely enhance the effects of eutrophication in the seagrass Posidonia oceanica? Frontiers in Marine Science **7**, 1–15.

**Pazzaglia J, Santillán-Sarmiento A, Ruocco M, Dattolo E, Ambrosino L, Marín-Guirao L, Procaccini G**. 2022*b*. Local environment modulates whole-transcriptome expression in the seagrass Posidonia oceanica under warming and nutrients excess. Environmental Pollution **303**, 119077.

**Pedersen O, Colmer TD, Borum J, Zavala-Perez A, Kendrick GA**. 2016. Heat stress of two tropical seagrass species during low tides - impact on underwater net photosynthesis, dark respiration and diel in situ internal aeration. New Phytologist **210**, 1207–1218.

**Pereda-Briones L, Terrados J, Tomas F**. 2019. Negative effects of warming on seagrass seedlings are not exacerbated by invasive algae. Marine Pollution Bulletin **141**, 36–45.

**Procaccini G, Dattolo E, Ruocco M**. 2023. Genetic diversity and connectivity of the Mediterranean seagrass Posidonia oceanica: state of art and future directions. Cah. Biol. Mar **64**, 105–114.

**Procaccini G, Ruocco M, Marín-Guirao L, *et al.*** 2017. Depth-specific fluctuations of gene expression and protein abundance modulate the photophysiology in the seagrass Posidonia oceanica. Scientific Reports **7**, 1–15.

**Ramajo L, Lagos NA, Duarte CM**. 2019. Seagrass Posidonia oceanica diel pH fluctuations reduce the mortality of epiphytic forams under experimental ocean acidification. Marine Pollution Bulletin **146**, 247–254.

**Re R, Pellegrini N, Proteggente A, Pannala A, Yang M, Rice-Evans C**. 1999. Antioxidant activity applying an improved ABTS radical cation decolorization assay. Free Radical Biology and Medicine **26**, 1231–1237.

**Reimer JJ, Thiele B, Biermann RT, Junker-Frohn L V., Wiese-Klinkenberg A, Usadel B, Wormit A**. 2021. Tomato leaves under stress: a comparison of stress response to mild abiotic stress between a cultivated and a wild tomato species. Plant Molecular Biology 2021 107:3 **107**, 177–206.

**Repolho T, Duarte B, Dionísio G, Paula JR, Lopes AR, Rosa IC, Grilo TF, Caçador I, Calado R, Rosa R**. 2017. Seagrass ecophysiological performance under ocean warming and acidification. Scientific Reports **7**, 1–12.

**Riccio-Rengifo C, Finke J, Rocha C**. 2021. Identifying stress responsive genes using overlapping communities in co-expression networks. BMC Bioinformatics **22**, 1–17.

**Rinaldi A, Martinez M, Badalamenti F, D’Anna G, Mirto S, Marín-Guirao L, Procaccini G, Montalto V**. 2023. The ontogeny-specific thermal sensitivity of the seagrass Posidonia oceanica. Frontiers in Marine Science **10**, 1–11.

**Robinson M, McCarthy D, Smyth G**. 2010. edgeR: a Bioconductor package for differential expression analysis of digital gene expression data. Bioinformatics (Oxford, England) **26**, 139–140.

**Rubio L, García-Pérez D, Davies JM, Fernández JA**. 2020. Short-Term Response of Cytosolic NO−3 to Inorganic Carbon Increase in Posidonia oceanica Leaf Cells. Frontiers in Plant Science **11**, 1.

**Ruiz JM, Marín-Guirao L, García-Muñoz R, *et al.*** 2018. Experimental evidence of warming-induced flowering in the Mediterranean seagrass Posidonia oceanica. Marine Pollution Bulletin **134**, 49–54.

**Ruocco M, Entrambasaguas L, Dattolo E, Milito A, Marín‐Guirao L, Procaccini G**. 2020. A king and vassals’ tale: molecular signatures of clonal integration in *Posidonia oceanica* under chronic light shortage. Journal of Ecology, 1365-2745.13479.

**Ruocco M, Marín-Guirao L, Procaccini G**. 2019*a*. Within- and among-leaf variations in photo-physiological functions, gene expression and DNA methylation patterns in the large-sized seagrass Posidonia oceanica. Marine Biology **166**, 24.

**Ruocco M, Marín L, Gabriele G**. 2019*b*. Within ‑ and among ‑ leaf variations in photo ‑ physiological functions , gene expression and DNA methylation patterns in the large ‑ sized seagrass Posidonia oceanica. Marine Biology **166**, 3–24.

**Ruocco M, Musacchia F, Olivé I, Costa MM, Barrote I, Santos R, Sanges R, Procaccini G, Silva J**. 2017. Genomewide transcriptional reprogramming in the seagrass Cymodocea nodosa under experimental ocean acidification. Molecular Ecology **26**, 4241–4259.

**Sandoval-Gil JM, Ruiz JM, Marín-Guirao L**. 2023. Advances in understanding multilevel responses of seagrasses to hypersalinity. Marine Environmental Research **183**, 105809.

**Santillán-Sarmiento A, Pazzaglia J, Ruocco M, Dattolo E, Ambrosino L, Winters G, Marin-Guirao L, Procaccini G**. 2023. Gene co-expression network analysis for the selection of candidate early warning indicators of heat and nutrient stress in Posidonia oceanica. Science of The Total Environment **877**, 162517.

**Scafaro AP, Fan Y, Posch BC, Garcia A, Coast O, Atkin OK**. 2021. Responses of leaf respiration to heatwaves. Plant Cell and Environment doi: 10.1111/PCE.14018.

**Shokat S, Novák O, Široká J, Singh S, Singh Gill K, Roitsch T, Großkinsky DK, Liu F**. 2021. Elevated CO 2 modulates the effect of heat stress responses in Triticum aestivum by differential expression of an isoflavone reductase-like gene. Journal of Experimental Botany **72**, 7594–7609.

**Silva J, Barrote I, Costa MM, Albano S, Santos R**. 2013. Physiological Responses of Zostera marina and Cymodocea nodosa to Light-Limitation Stress. (DA Campbell, Ed.). PLoS ONE **8**, e81058.

**Stipcich P, La Manna G, Ceccherelli G**. 2024. Warming-induced flowering and fruiting in the seagrass Posidonia oceanica and uncertainties due to context-dependent features. Marine Biology **171**, 1–12.

**Stipcich P, Pansini A, Beca-Carretero P, Stengel DB, Ceccherelli G**. 2022*a*. Field thermo acclimation increases the resilience of Posidonia oceanica seedlings to marine heat waves. Marine Pollution Bulletin **184**, 114230.

**Stipcich P, Stipcich P, Marín-guirao L, Pansini A, Pinna F, Fraser MW**. 2022*b*. Effects of Current and Future Summer Marine Heat Waves on Posidonia oceanica: Plant Origin Matters? **4**, 844831.

**Tittensor DP, Mora C, Jetz W, Lotze HK, Ricard D, Berghe E Vanden, Worm B**. 2010. Global patterns and predictors of marine biodiversity across taxa. Nature **466**, 1098–1101.

**Touchette BW, Burkholder JM**. 2000. Review of nitrogen and phosphorus metabolism in seagrasses. Journal of Experimental Marine Biology and Ecology 250 **250**, 133–167.

**Traboni C, Mammola SD, Ruocco M, Ontoria Y, Ruiz JM, Procaccini G, Marín-Guirao L**. 2018. Investigating cellular stress response to heat stress in the seagrass Posidonia oceanica in a global change scenario. Marine Environmental Research **141**, 12–23.

**Tutar O, Marín-Guirao L, Ruiz JM, Procaccini G**. 2017. Antioxidant response to heat stress in seagrasses. A gene expression study. Marine Environmental Research doi: 10.1016/j.marenvres.2017.10.011.

**Vanlerberghe GC**. 2013. Alternative oxidase: A mitochondrial respiratory pathway to maintain metabolic and signaling homeostasis during abiotic and biotic stress in plants. Int J Mol Sci.

**Viana IG, Artika SR, Moreira-Saporiti A, Teichberg M**. 2023. Limited trait responses of a tropical seagrass to the combination of increasing pCO2 and warming. Journal of Experimental Botany **74**, 472–488.

**Wang Y ge, He X, Ma W ying, Zhao X qiang, Li B, Tong Y ping**. 2014. Wheat PROTON GRADIENT REGULATION 5 is involved in tolerance to photoinhibition. Journal of Integrative Agriculture **13**, 1206–1215.

**Williams EJB, Bowles DJ**. 2004. Coexpression of Neighboring Genes in the Genome of Arabidopsis thaliana. Genome Research **14**, 1060–1067.

**Xi Y, Park SR, Kim DH, Kim ED, Sung S**. 2020. Transcriptome and epigenome analyses of vernalization in Arabidopsis thaliana. The Plant journal : for cell and molecular biology **103**, 1490.

**York PH, Gruber RK, Hill R, Ralph PJ, Booth DJ, Macreadie PI**. 2013. Physiological and Morphological Responses of the Temperate Seagrass Zostera muelleri to Multiple Stressors: Investigating the Interactive Effects of Light and Temperature. PLoS ONE **8**.

**Yu B, Liu J, Wu D, Liu Y, Cen W, Wang S, Li R, Luo J**. 2020. Correction to: Weighted gene coexpression network analysis-based identification of key modules and hub genes associated with drought sensitivity in rice (BMC Plant Biology, (2020), 20, 1, (478), 10.1186/s12870-020-02705-9). BMC Plant Biology **20**, 1–21.

**Zayas-Santiago C C, Rivas-Ubach A, Kuo L J, Ward N D, Zimmerman R C** 2020. Metabolic profiling reveals biochemical pathways responsible for eelgrass response to elevated CO_2_ and temperature. Scientific Reports, 10(1), 4693.

**Zemach A, Kim MY, Hsieh PH, Coleman-Derr D, Eshed-Williams L, Thao K, Harmer SL, Zilberman D**. 2013. The arabidopsis nucleosome remodeler DDM1 allows DNA methyltransferases to access H1-containing heterochromatin. Cell **153**, 193–205.

**Zhang B, Horvath S**. 2005. A general framework for weighted gene co-expression network analysis. Statistical Applications in Genetics and Molecular Biology **4**.

**Zinta G, Abdelgawad H, Domagalska MA, Vergauwen L, Knapen D, Nijs I, Janssens IA, Beemster GTS, Asard H**. 2014. Physiological, biochemical, and genome-wide transcriptional analysis reveals that elevated CO2 mitigates the impact of combined heat wave and drought stress in Arabidopsis thaliana at multiple organizational levels. Global change biology **20**, 3670–3685.
